# Exploring the mobility in the Madrid Community

**DOI:** 10.1038/s41598-023-27979-5

**Published:** 2023-01-17

**Authors:** Mary Luz Mouronte-López, Javier Gómez

**Affiliations:** 1 Higher Polytechnic School, Universidad Francisco de Vitoria, Carretera Pozuelo a, Av de Majadahonda, Km 1.800, 28223 Madrid, Spain; 2 Grupo de Sistemas Complejos, Escuela Técnica Superior de Ingeniería Agronómica, Alimentaria y de Biosistemas, Universidad Politécnica de Madrid, Avda. Puerta de Hierro 2-4, 28040 Madrid, Spain

**Keywords:** Complex networks, Scientific data

## Abstract

Displacements within urban spaces have attracted particular interest among researchers. We examine the journeys that happen in the Madrid Community considering 24 travel typologies and 1390 administrative areas. From an origin–destination (*OD*) matrix, four classes of major flows are characterised through coarse-graining: hotspot–non-hotspots, non-hotspot–hotspots, hotspots–hotspots, non-hotspot–non-hotspot. In order to make comparisons between them with respect to spatial and temporal patterns, several statistical tests are performed. The spatial activity as well as transition probabilities between administrative zones are also analysed. The mobility network’s topology is examined (some parameters such as maximal connected components, average degree, betweenness, and assortativity as well as the k-cores are checked). A model describing the formation of links between zones (existence of at least one trip between them) is constructed based on certain measures of affinity between areas.

## Introduction

Methods to extract a coarse-grained signature of the urban mobility networks have been suggested^1^, as well as procedures for dissecting the urban spatial structure^2–5^ from human mobility patterns. The public transport networks have been thoroughly analysed^6–10^ and so too have their travel demands^11^. The shared mobility services have also been investigated^12–14^.

In particular, several coarse-graining methods have been applied to study certain mobility networks. A non-parametric clustering algorithm was utilised to separate nodes into hotspot and non-hotspot classes referred to taxi mobility data from New York and Chicago^11^. An origin–destination (*OD*) matrix with nodes denoting city locations and weighted edges representing the number of trips between them, was also augmented with other node attributes (such as socio-economic features). This is done in order to study the spatio-temporal characteristics of hotspots in relation to different types of socio-economic activities^15^. Various methods to estimate the *OD* matrix have also been proposed (data obtained from surveys^16^, travel diaries^17^, or vehicle identification systems^18–20^, as well as from mobile phone or GPS based movement evidence^11,21^. An examination of the transition probabilities has been utilised to disentangle the human mobility patterns in several contests^22,23^.

Researchers have studied several aspects of mobility in Madrid (community or city). The pieces of investigation have performed a comparative analysis on university student mobility to obtain statistics on the basis of which methods/modes of transport need to focus on the reduction of CO2 emissions^24^. The spatial and temporal trip behaviours in BiciMAD (a Bike-Sharing System in Madrid city) were explored^25^ from 21 million GPS records and various maps. Research^1^ suggested a procedure to extract a coarse-grained signature of certain mobility networks, from an *OD* matrix in which each $F_{ij}$ element symbolised the number of persons living in location *i* and commuting to location *j* in which they had their main activity (work or school). In this way a 2 $\times$ 2 matrix was generated consisting of four flow categories: Integrated (I), Convergent (C), Divergent (D) and Random (R). Integrated flows (I) are those displacements that go to and from residential hotspots, Convergent flows (C) are those movements that go from random residential places to work hotspots; Divergent flows (D) are those trips that go from residential hotspots to places of random activity; and finally, Random flows (R) are those displacements moving to and from places that are not hotspots. From this characterization as well as by using mobile phone records collected during a 5-week period, the entire mobility networks of 31 Spanish urban areas were analysed. A clustering of metropolises considering the commuting patterns structure was made^1^ demonstrating that the mobility structure of some cities was not randomly organised. Using a similar method to^11^, we describe through a coarse-graining representation the 1390 administrative areas of the Madrid Community (a much larger area than that corresponding to Madrid city) as hotspot and non-hotspot zones considering 24 types of displacements. Various commonalities and differences were detected between flows (velocities, distances, and temporal patterns). The most probable lengths and durations of displacements between zones are also analysed. Additionally, the 24 mobility networks corresponding to the diverse modes of displacements are studied using the networks theory^26^. A model that correctly reproduces the formation of links (trips) based on local, quasi-local and global metrics is also built.

## Results

The mobility of the inhabitants of the Madrid Community is explored from a household survey conducted by the Regional Transport Consortium of Madrid^27^, (see “Methods” section). As a result, we have 222,744 trips with their origins and destinations, both corresponding to the 1390 administrative transport zones into which Madrid Community is divided.

### Characterization of the displacements

#### Reasons, trip modes and trip start times

According to the analysed data set, the motives for the trip with the four highest percentages were Work (25.68%), Study (14.99%), Personal Business (12.59%) and Shopping (12.35%) (see the Supplementary Material Document). The most highly used travel mode categories were Sport/Walking, Driver in private car, Subway, Urban bus, Commuter trains as well as Intercity bus (see Table 1).

**Table 1:** Number and percentage of displacements by priority transport type.

Type of primary transport used for the trip	Number	Percentage (%)	Type of primary transport used for the trip	Number	Percentage (%)
T1: **Commuter trains**	**8955**	**4.02**	T13:Driver or passenger in rented car without driver	92	0.04
T2: Intercity bus	9325	4.19	T14: Passenger in private car	20,571	9.24
T3: Urban bus other municipality	1869	0.84	T15: Passenger in company car	303	0.14
T4: **Subway**	**19,057**	**8.56**	T16: Passenger in rented car with driver	52	0.02
T5: Light subway	247	0.11	T17: Private motorbike	1805	0.81
T6: **Urban bus**	**13,773**	**6.18**	T18: Public motorbike	5	0
T7: Rest of trains	99	0.04	T19: Company motorbike	13	0.01
T8: Discretionary bus	1106	0.50	T20: Private bicycle	872	0.39
T9: Long-distance bus	38	0.02	T21:Public bicycle	74	0.03
T10: Taxi	1033	0.46	T22: Company bicycle	2	0
T11: **Driver in private car**	**66,036**	**29.65**	T23: Other	728	0.33
T12: Driver in company car	1847	0.83	T24:**Walking**	**74,842**	**33.60**

Significant values are in [bold].

The histograms as well the cumulative probability distributions corresponding to the trip start times by travel type were calculated. In order to compare them, the Kolmogorov–Smirnov test^28^ was applied with a significance level equal to 0.05. For each pairwise comparisons $(T_{i}, T_{j})$, for $j \not = i$, and *i*, *j* varying 1, 2, ...24, the number of times in which *p* value was less than 0.05 was counted, finding that those trip types that showing the greatest differences to the rest were in order T8 (Discretionary bus), T19 (Motorcycle/motorcycle company), T15 (Passenger in company car), and T12 (Driver in company car). Some analogies (*p* value $\ge 0.05$) were identified between T4–T6, T4–T14, T4–T20, T4–T24, T6–T14, T6–T20, T6–T24, T14–T20, T14–T24. Consequently, types T4 (Subway), T6 (Urban bus), T14 (Passenger in private car), T20 (private bicycle) and T24 (walking) presented analogies with respect to their journey start times cumulative probability distributions. The rest of the trip types did not show any commonalities with each other, which points to the existence of connections between the travel mode and the departure time choices (see the Supplementary Material Document).

#### Trip distances

Taking as a reference the transport zones in which the Madrid Community is divided. An *OD* matrix was generated, in which each element $T_{ij}$ symbolises the number of trips from zone (i) to zone (j)^29^. For each $i= 1, 2,\ldots , 1390$, various parameters were established:

$in-degree$ $k_{i}^{in}$, which symbolises the total number of trips arriving to zone *i*. It can be defined as^30^:

1 $$\begin{aligned} k_{i}^{in}=\sum _{l=1}^{N}T_{li} \end{aligned}$$

where $T_{li}$ is the number of journeys from *l* area to *i* area.

$out-degree$ $k_{i}^{out}$, which indicates the total number of trips leaving zone *i*. It can be defined as^30^:

2 $$\begin{aligned} k_{i}^{out}=\sum _{l=1}^{N}T_{il} \end{aligned}$$

where $T_{il}$ is the number of journeys from *i* area to *l* area. The calculation of the *OD* matrix was done globally and for each of the 24 trip types.

**Figure 1:**
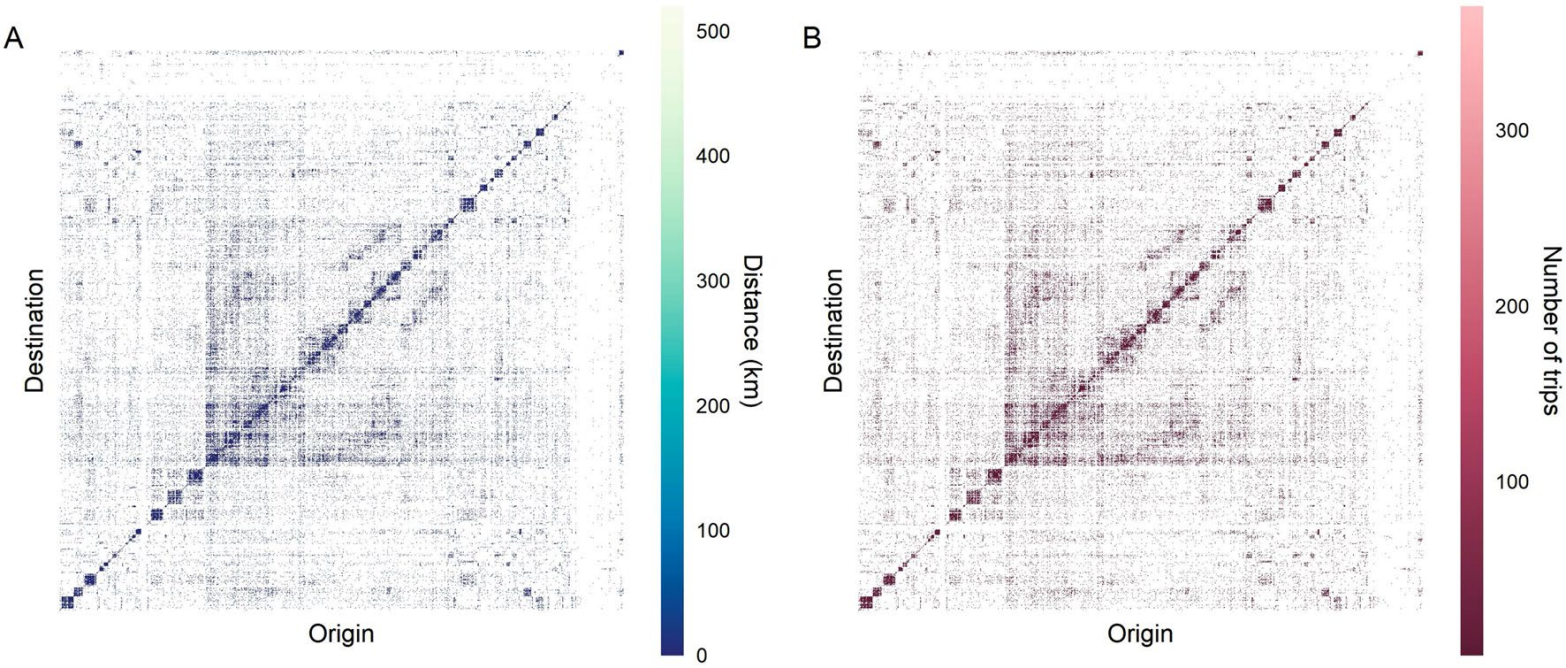
Overall inter-zone activity for all trip types (**A** *D* matrix, **B** *OD* matrix).

A distance matrix (*D*) was also constructed where each element $d_{ij}$ symbolised the geographical distance in kilometres between the zones *i* and *j*, for $i,j = 1, 2, 3, \ldots 1390$. Figure 1 presents the *OD* and *D* matrices for all types of trip. If we relate the data of the *OD* matrix with those of the *D* matrix, it can be observed that a relevant number of trips correspond to distances below 100 km. A figure showing for each *i*, both $k_{i}^{in}$ and $k_{i}^{out}$ the probability distributions has also been included in the Supplementary Material Document.

Using the *OD* matrix, it was possible to analyse mobility in the Madrid Community as a dynamic process, establishing the transition probability between administrative zones according to the travel distance, which can be defined as:

3 $$\begin{aligned} w_{i\rightarrow j}^{(out)}=\frac{T_{ij}}{k_{i}^{(out)}} \end{aligned}$$

where the following condition is fulfilled:

4 $$\begin{aligned} \sum _{l=1}^{N}w_{i\rightarrow j}^{(out)}=1 \end{aligned}$$

**Figure 2:**
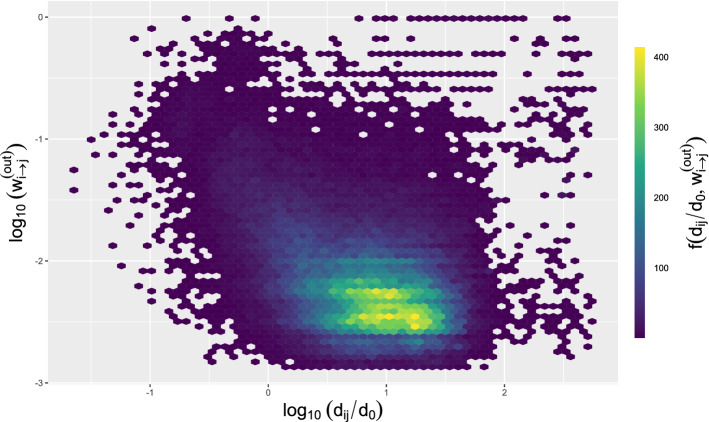
Number of pairs ($log_{10} w_{i\rightarrow j}^{(out)}$, $log_{10} (\frac{d_{ij}}{d_{0}})$) for all trip types in the Madrid Community.

Each geographical distance $d_{ij}$, which is the *i*,*j* elem of the *D* matrix, corresponds to transition probability $w_{i\rightarrow j}^{(out)}$. Figure 2 shows the number of pairs ($log_{10} w_{i\rightarrow j}^{(out)}$, $log_{10} (\frac{d_{ij}}{d_{0}})$) for all trip types, where all longitudes have been normalised as $d_{ij}/d_{0}$, $d_{0}=1$ km^30^. The most likely displacements correspond to spaces between 5 and 30 km. This is in accordance with Fig. 6, which shows that not many journeys between far areas existed. The information corresponding to each trip mode has been included in the Supplementary Material Document.

Table 2 depicts the most frequent travel distances range (calculated as $log_{10} (\frac{d_{ij}}{d_{0}})$) as well as the transition probabilities range (computed as $log_{10} w_{i\rightarrow j}^{(out)}$). The most likely distances were between 1.91 and 26.30 km both for public transport and private cars. Among public transport, Urban bus and Subway exhibited the lowest value showing that they are commonly utilised for short journeys (many trips could be done on foot). Walks were also mostly short in length.

**Table 2:** The most frequent travel distances range (calculated as $log_{10} (\frac{d_{ij}}{d_{0}})$) and the transition probabilities (estimated as $log_{10} w_{i\rightarrow j}^{(out)}$): (T1) Train, T2) Interurban bus, (T4) Subway, (T6) Urban bus, (T11) Driver in private driver car, (T14) Passenger in private car, (T24) Walking.

Type of transport	$log_{10} (\frac{d_{ij}}{d_{0}})$	$log_{10} w_{i\rightarrow j}^{(out)}$	$(\frac{d_{ij}}{d_{0}})$	$w_{i\rightarrow j}^{(out)}$
(T1): Commuter trains	0.95:1.42	− 2.37 : − 2.54	8.91:26.30	$4.27\times 10^{-3}:2.897\times 10^{-3}$
(T2) Intercity bus	0.91:1.40	− 2.43 : − 2.46	8.13:25.12	$3.72\times 10^{-3}:3.72\times 10^{-3}$
(T4) Subway	0.38 : 0.94	− 2.28 : − 2.51	2.40:8.71	$5.24\times 10^{-3}:3.09\times 10^{-3}$
(T6) Urban bus	0.28:0.71	− 2.25 : − 2.50	1.91:5.13	$5.62\times 10^{-3}:3.16\times 10^{-3}$
(T11) Driver in private car	0.87:1.28	− 2.25 : − 2.53	7.41:19.05	$5.62\times 10^{-3}:2.95\times 10^{-3}$
(T14) Passenger in private car	0.31:1.19	− 2.45 : − 2.50	2.04:15.49	$3.555\times 10^{-3}:3.16\times 10^{-3}$
(T24) Walking	− 0.22:0.40	− 1.75 : − 2.45	0.60:2.51	$17.78\times 10^{-3}:3.55\times 10^{-3}$

The median of the displacements was also estimated, where (T18) Public motorbike, (T24) Walking, and (T3) Urban bus other municipality types exhibited the lowest median (< 2 km). The highest values corresponded to (T2) Intercity bus, (T1): Commuter trains, (T7) Rest of trains and (T9) Long-distance bus types (> 20 kilometres). We also calculated the mean distances but a high standard deviation was found (see the Supplementary Material Document).

#### Trip duration

**Figure 3:**
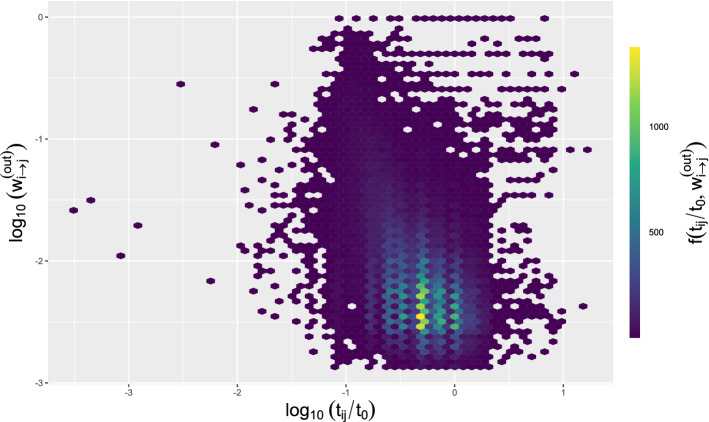
Number of pairs ($log_{10} w_{i\rightarrow j}^{(out)}$, $log_{10}(\frac{t_{ij}}{t_{0}})$) for all trip types in the Madrid Community.

Analogously to the travel distances explored in the previous section, the travel duration was examined. Figure 3 displays the number of pairs ($log_{10} w_{i\rightarrow j}^{(out)}$, $log_{10}(\frac{t_{ij}}{t_{0}})$) for all trip types, being the journey duration normalised as $t_{ij}/t_{0}$ where $t_{0}=1$ h. The highest probabilities happened around 0.40 h ($log_{10} w_{i\rightarrow j}^{(out)}=-2.5$). The data corresponding to each trip mode have been incorporated in the Supplementary Material Document. Table 3 shows the top frequent travel time range (estimated as $log_{10} (\frac{t_{ij}}{t_{0}})$) and the transition probabilities range (calculated as $log_{10} w_{i\rightarrow j}^{(out)}$).

Regarding the median of the journey time, the private car was frequently used for journeys of a shorter time period. The displacement as a passenger (T14) showed the lowest value (10 min) and as a driver (T11) also exhibited a very close magnitude. The trip types (T3) Urban bus other municipality, (T5) Light subway, (T15) Passenger in company car, (T18) Public motorbike, (T19) Company motorbike, and (T24) Walking presented a slightly higher median with a value equal to 15 min. Analogy between the duration of trips made on foot with those made in other motorised vehicles with more similar likely distances, showed that these journeys could have been made by foot without much impact on trip length. The highest values (60 min) corresponded to (T1) Commuter trains, (T7) Rest of trains and (T9) Long-distance bus (see the Supplementary Material Document).

**Table 3:** The most frequent travel time range (calculated as $log_{10}(\frac{t_{ij}}{t_{0}})$ and the transition probabilities (estimated as $log_{10} w_{i\rightarrow j}^{(out)}$): (1) Commuter trains, (2) Intercity bus, (4) Subway, (6) Urban bus, (11) Driver in private car, (14) Passenger in private car, (24) Walking.

Type of transport	$log_{10}(\frac{t_{ij}}{t_{0}})$	$log_{10} w_{i\rightarrow j}^{(out)}$	$(\frac{t_{ij}}{t_{0}})$	$w_{i\rightarrow j}^{(out)}$
(T1): Commuter trains	− 0.13:0.17	− 2.45 : − 2.53	0.74:1.47	$3.55\times 10^{-3}:2.95\times 10^{-3}$
(T2) Intercity bus	0.02:0.18	− 2.24 : − 2.50	1.05:1.51	$5.75\times 10^{-3}:3.16 \times 10^{-3}$
(T4) Subway	− 0.29:− 0.13	− 2.28 : − 2.51	0.51:0.74	$5.24\times 10^{-3}:3.09\times 10^{-3}$
(T6) Urban bus	− 0.47:− 0.30	− 1.95 : − 2.50	0.34:0.50	$10.00\times 10^{-3}:3.16\times 10^{-3}$
(T11) Driver in private car	− 0.60:− 0.32	− 2.16 : − 2.53	0.25:0.48	$6.92\times 10^{-3}:2.95\times 10^{-3}$
(T14) Passenger in private car	− 0.60:− 0.29	− 2.21 : − 2.50	0.25:0.51	$3.17\times 10^{-3}:3.16\times 10^{-3}$
(T24) Walking	− 0.59:− 0.28	− 2.25 : − 2.50	0.26:0.52	$5.62\times 10^{-3}:3.16\times 10^{-3}$

#### Characterisation of the major flows

Based on the *OD* matrix, all administrative areas into which the Madrid Community is divided were characterised as hotspot or non-hotspot^31^. Thus, four types of fluxes (displacements) between zones were established: *HH* (origin: hotspot, destination: hotspot), *HN* (origin: hotspot, destination: non-hotspot), *NH* (origin: non-hotspot, destination: hotspot) and *NN* (origin: non-hotspot, destination: non-hotspot)^32,33^. The number of trips made in the $[h_{l}$, $h_{l+1}]$ interval, for $l= 0, 1,\ldots , 23$, where $h_{l}$ symbolises the 1 h in a day, was computed. The correlations between the types of fluxes were calculated through the Spearman’s method, values $\ge 0.94$ were obtained. The normality of distributions was tested applying the Anderson–Darling test^28^ with a significance level equal to 0.05. Because the obtained p value was < 0.05, the Spearman’s method was utilised for the calculation. According to Fig. 5 (in the graphical representation, those trips that happened in $[h_{l}$, $h_{l+1}]$ were assigned to the value *l*), all flows presented a maximum at 8:00 a.m. The *HH* movement exhibited two maximum values at 14:00 and 17:00, respectively), while the rest of the flows also showed two maximum values at the aforementioned times. The *HH* flux presented a greater number of trips over all others, while the *HN* and *NH* movements exhibited very similar magnitudes, with one slightly prevailing over the other by time of day. Table 4 presents for the aforementioned times, displaying the reason for the displacements, corresponding the highest proportions to Work and Study causes.

**Figure 4:**
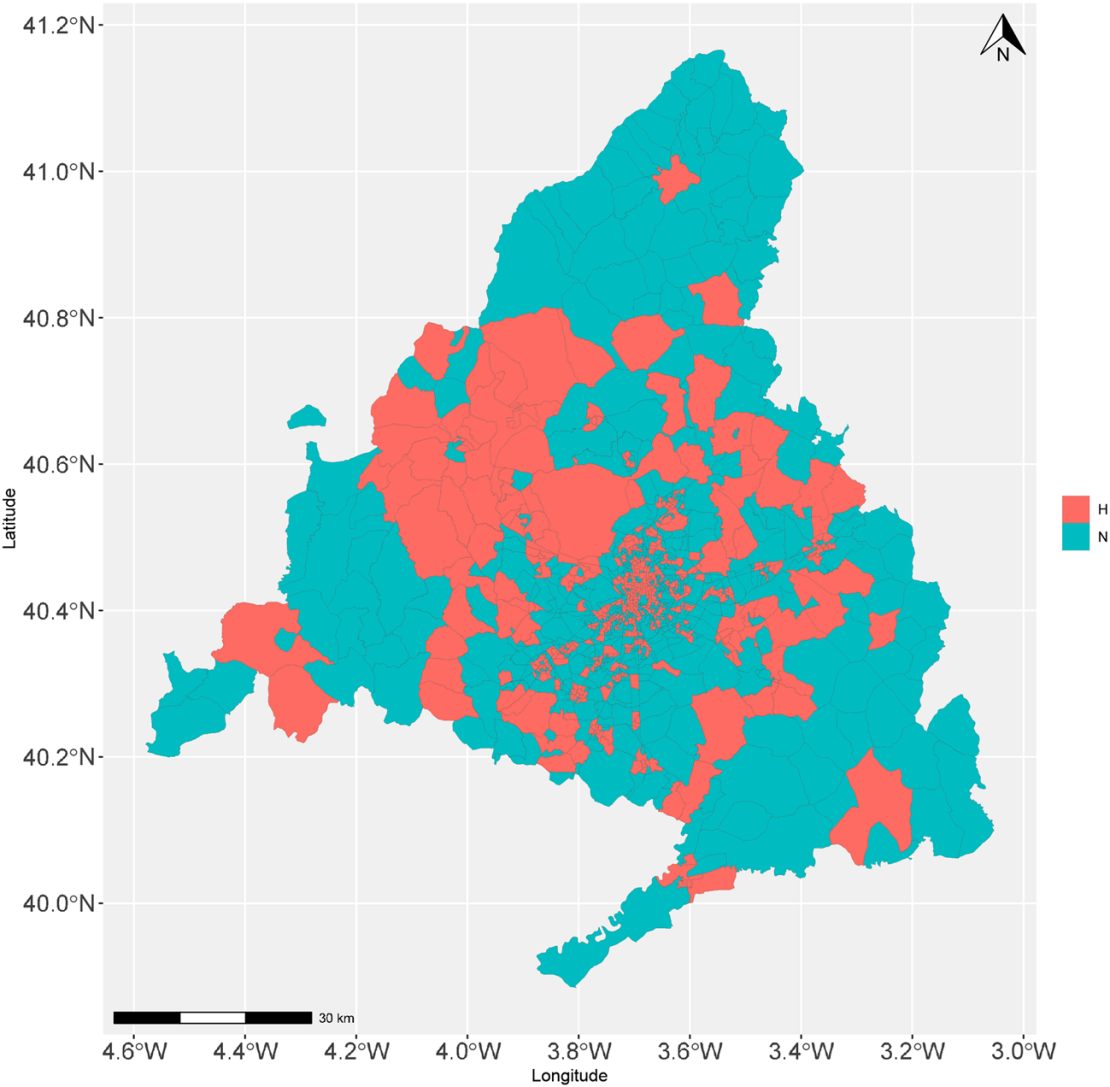
Map categorising the zones per origin. Information retrieved from^34^ was utilised for the construction of the map.

**Table 4:** At 08:00 and at [14:00, 17:00] interval, percentage of displacements by priority reason for trip.

Priority reason for trip	At 08:00 (%)	At [14:00, 17:00] (%)	Priority reason for trip	At 08:00 (%)	At [14:00, 17:00] (%)
Home	0.23	0.38	Accompanying another person	16.58	12.19
**Work**	**30.72**	**27.05**	Leisure	0.74	4.73
Work management	1.72	1.84	Sport/walking	2.99	6.42
Study	**39.05**	**22.57**	Personal business	4.17	13.21
Shopping	0.82	5.87	Other address	0.25	1.27
Medical	2.41	3.72	Other	0.32	0.74

Significant values are in [bold].

Figure 4 shows the map of the Madrid Community divided into hotspots and non-hotspots zones. It can be observed that a higher concentration of hotspot areas exist in the centre of the community, while most of the non-hotspot areas are distributed on the periphery, which responds to a model of the large metropolis with nearby dormitory towns^35^. 543 hotspots and 841 non-hotspots were identified for origins and 539 hotspots and 833 non-hotspots for destinations (a map showing the 5 areas with the highest number of trips has been included in the Supplementary Material Document).

**Figure 5:**
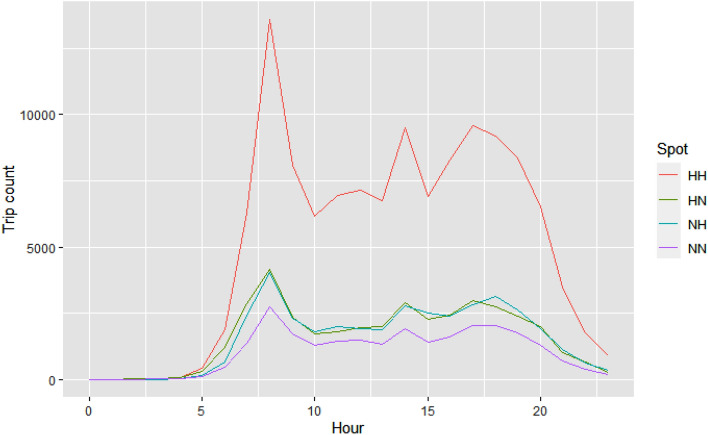
Temporal average number of hourly trips within different flows.

**Figure 6:**
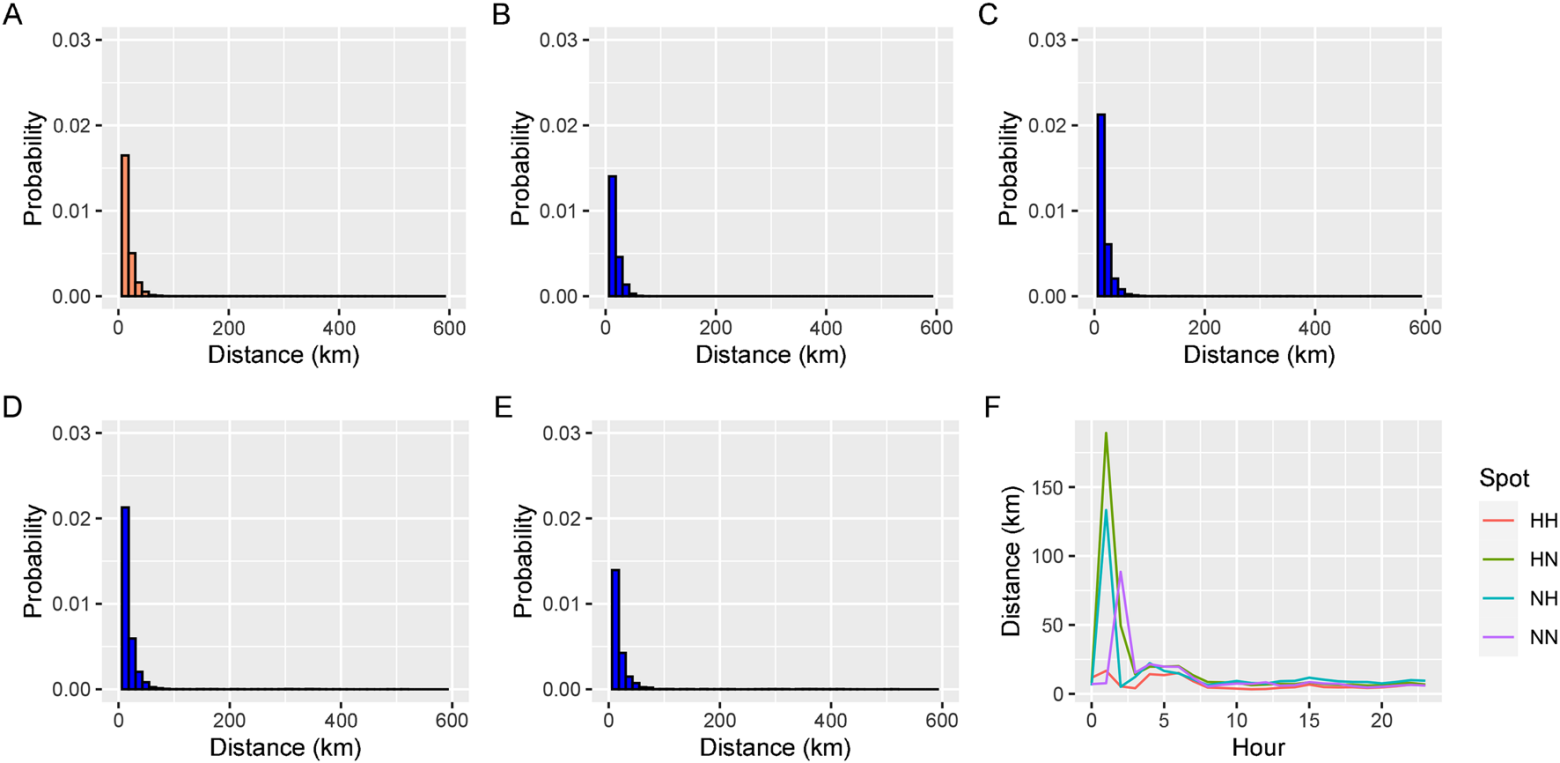
Trip distance distribution and travel distance by time of day for HH, NH, HN, and NN flow types.

The highest correlations (= 0.99) were found between *HH* and *NN* as well as *HN* and *NH* fluxes (see the Supplementary Material Document). 16 areas varied their hotspot or non-hotspot status depending on whether they were the origins or destinations, while the rest of the 1390 areas maintained their role. Thus, most of the *HN* and *NH* displacements happened between different zones; however, we can not know from *H* or *N* role, whether *HH* and *NN* movements were inter-zone or intra-zone.

**Figure 7:**
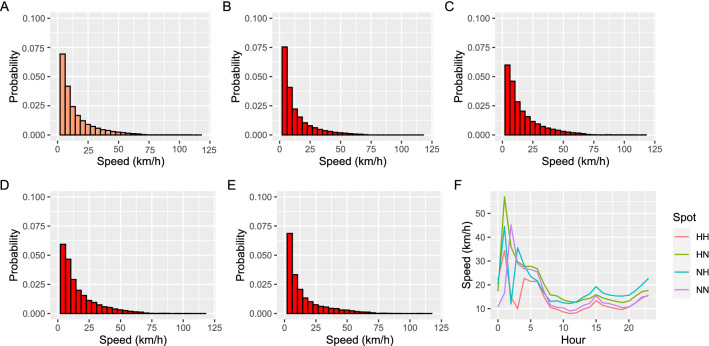
Speed distance distribution and trip speeds by time of day for HH, NH, HN, and NN flow.

Analogously to what was carried out in the “Trip start times” section, we compared the cumulative probability distributions by type of flow. In terms of travel distances some similarity was identified between *HN* and *NH* flows (*p* value $\ge 0.05)$. Various differences were also detected between *HN* and *NH*, as well as between *HH* and *NN* displacements in relation to velocity (*p* value $<0.05$). Spanish Law sets a speed limit of 120 km/h, so trips over that speed were removed. The highest recorded velocity by a human running is around 44 km/h^36^, so velocities below this value were removed. The travel distance as well as the achieved speed in the $[h_{l}$, $h_{l+1}]$ interval, $l = 0, 1, \ldots 23$ are also shown in Figs. 6 and 7. With reference to velocity, a high level of similarity existed between the shape of the flow charts. Higher-speed and higher-distance of travel happened at night for all fluxes, with the exception of the *HH* movements, displayed stable results in the distance travelled during the day.

### Topological characterisation of the mobility networks.

#### Structural analysis

The mobility networks (those determined by the primary type of trip), were represented as a graph $G = (NS;LS)$, where, *NS* is the set of nodes, each one symbolising the existing administrative zones, and *LS* is the set of links between them. An adjacency matrix of $N \times N$ dimension *A*(*G*) was built as a bi-dimensional representation of the relationships between nodes, where $A_{ij} = 1$ when a connection between nodes *i* and *j* exists and $A_{ij} = 0$ otherwise. *N* represents the number of nodes in *NS*. All redundant links, as well as ties were eliminated in *G*.

The connectivity of the networks was examined calculating the number of maximal weakly connected subgraphs in each *G*. T1 (commuter trains), T4 (subway), T11 (private car driver), T14 (private passenger car) and T18 (Public motorcycle/moped) networks were fully connected, while three components were detected in the whole network. No strongly connected subgraphs were found. We also calculate the largest weakly connected subgraph (giant component *GC*), in order to examine the main structural properties of the networks (see the Supplementary Material Document).

The average shortest path (*asp*) exhibited values in the [1, 8.56] interval. The highest magnitudes corresponded to T12 (driver in company car) and T20 (private bicycle), respectively. T7 (other train types), T16 (passenger in rental car with driver), T18 (public motorcycle/moped), T19 (motorcycle/moped company), and T22 (other transportation) showed the lowest values. The Networks with a low <asp> and many nodes provide a high-level communication between zones.

The average degree $<k>$ was in the [1, 65.48] range. Its largest values occurred in type T11 (driver in private car ), T4 (subway), and T24 (walking). A higher $<k>$ a higher average accessibility is provided by the mobility network. Considering all types of trips, $<k>$ and $<asp>$ were equal to 16.11, and 2.31.

The cumulative probability distributions focusing on stops which include main types of public transport (Commuter trains, Intercity buses, Urban buses, Subway and Light subway) by area were also explored. The Kolmogorov–Smirnov test^28^ was applied, similarly to what was calculated in Trip start times. Some analogy was detected between Subway and Commuter trains (*p* value $\ge 0.05$), while the rest of trip typologies did not exhibit any similarity (see the Supplementary Material Document). However, although there are similarities between the distributions of both network’s stops by zones, the $<k>$ of the Subway is more than twice as high as the figure related to Commuter trains.

All mobility networks showed negative assortativity except type T2 (Interurban bus), T6 (Urban bus), T11 (driver in private car), T14 (passenger in private car) and T24 (walking) networks. Not distinguishing by type of travel, the network exhibited a very low positive assortativity (0.06). Networks with low assortativity are not very robust with respect to failures in high-degree nodes and they present a low vulnerability to random failures in nodes. Therefore, a public transport design that not only integrates networks with low and high assortativity, but also compensates their effects could be an appropriate option.

According to the k-core decomposition^37^, the whole network showed a high hierarchy with $k_{Max}-core=103$. In those networks corresponding to the public transport network, the nodes with the highest $k_{Max}-core$ symbolise the most accessible and transferable capacity areas. (T1): Commuter trains, (T2) Intercity bus, (T4) Subway, and (T6) Urban bus exhibited the highest $k_{Max}-core$ (> 10), with a percentage of nodes in $k_{Max}-core$ (> 10%). Regarding zones with disabled access, the networks (T1) Commuter trains, (T2) Intercity bus, (T3) Urban bus other municipality, (T4) Subway, which had both high $k_{Max}-core$ and number of nodes in the lower k-shells (Data related to all mobility network can be found in the Supplementary Material Document) presented a relevant level of protection against random disabled zones (random attacks)^38^.

A low betweenness was identified in all types of networks (< 0.06), except for T15 (Passenger in company car), T18 (Public motorbike/moped) and T19 (Motorcycle/motorcycle company). Networks exhibiting a low betweenness are more robust to failures than those with a high value^39,40^, which is particularly relevant in the public transport mobility networks.

Based on the matrix $W^{(out)}$ certain information about the mobility networks can be obtained. Similarly to^30^, we can rank the importance of each node. Although the $W^{(out)}$ matrix is not symmetric, its eigenvalues and eigenvectors provide relevant information. For the $W^{(out)}$ matrix, a left eigenvector, which is defined as a row vector $\vec {\phi _{L}}_{j}$ exists, if the following condition is satisfied:

5 $$\begin{aligned} \vec {\phi _{L}}_{j}W^{(out)}=\lambda _{L_{j}}\vec {\phi _{L}}_{j} \;\;\; for \;\;\; j=1,2,\ldots ,N \end{aligned}$$

where $\lambda _{L_{j}}$ are the associated eigenvalues to $\vec {\phi _{L}}_{j}$. The left eigenvector associated with the eigenvalue $\lambda _{L_{j}} = 1$ defines a ranking vector $\vec {P}^{\infty }$ with elements ${P}^{\infty }_{i}$ con $i=1,2,\ldots ,N$ and fulfils $(\vec {P}^{\infty }W^{(out)}=\vec {P}^{\infty })$, therefore:

6 $$\begin{aligned} \sum _{l=1}^{N}{P}_{l}^{\infty }w_{l\rightarrow j}^{(out)}={P}_{j}^{\infty } \end{aligned}$$

**Figure 8:**
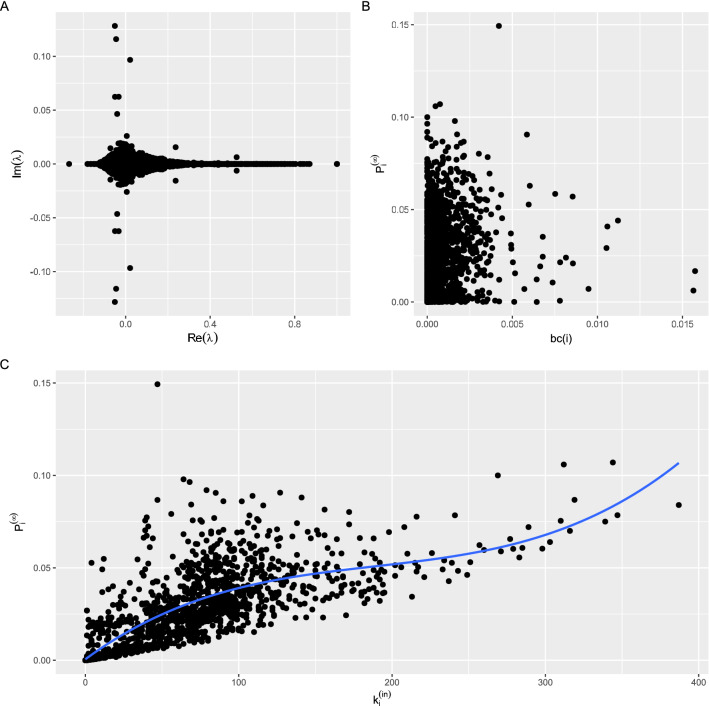
(**A**) eigenvalues $\lambda$ of $W^{(out)}$ fulfilling Eq. (6), (**B**) components ${P}^{\infty }_{i}$ of the eigenvector $\vec {P}^{\infty }$ with eigenvalue $\lambda = 1$ as a function of *bc*(*i*), (**C**) components ${P}^{\infty }_{i}$ of the eigenvector $\vec {P}^{\infty }$ with eigenvalue $\lambda = 1$ as a function of $k_{i}^{(in)}$.

Three $\lambda _{L_{j}}$ exist with a value equal to 1 (the highest eigenvalue $\lambda _{L_{j}}$ is included in the Supplementary Material Document for all mobility networks). In the study of random walks on networks, the vector $\vec {P}^{\infty }$ is the stationary probability distribution. The value ${P}^{\infty }_{i}$ is the probability of a random walker reaching the node *i* after a large number of steps^30^. In the analysis of mobility with a transition matrix $W^{(out)}$, the vector $\vec {P}^{\infty }$ establishes a ranking of the zones utilised in the definition of the origin-destination matrix. Those zones exhibiting a higher probability can be considered more important in the mobility network. Figure 8 shows the numerical values of ${P}^{\infty }_{i}$ according to their degree $k_{i}$ for $i = 1, 2,\ldots , N$.

Regarding components ${P}^{\infty }_{i}$ of the eigenvector $\vec {P}^{\infty }$ with eigenvalue $\lambda = 1$ as a function of $k_{i}^{(in)}$, we have carried out a fit polynomial regression model of degree 4, with a *R*-squared = 0.56950. The equation of the curve is as follows:

7 $$\begin{aligned} y = -3.180e-12 x^{4} + 6.780e-09 x^{3} -3.091e-06 x^{2} + 6.303e-04 x + 3.762e-04 \end{aligned}$$

The obtained *R*-squared for the polynomial regression fitting from degrees from 1 until 4 has been included in the Supplementary Material Document. No better results were achieved for higher degrees. The correlation between $\vec {P}^{\infty }$ and $k_{i}^{(in)}$, was computed utilising the Spearman’s Method, a value equal to 0.80531 was obtained.

Correlations between betweenness centrality and degree have been frequently examined, having shown different results by the research^41,42^. We calculated this correlation using the Spearman’s method, which was 0.252507 (the normality of the distributions was checked, similarly to what was carried out in the “Trip start times” section). We were also unable to find an appropriate fit polynomial regression model for components ${P}^{\infty }_{i}$ of the eigenvector $\vec {P}^{\infty }$ with eigenvalue $\lambda = 1$ as a function of *bc*(*i*).

#### Inter-zone links formation

The Generalised Linear Model (*GLM*) has been shown to appropriately reproduce the formation of physical links between nodes in public transport networks^8^. We examined whether this model could also adequately describe the link formation process between zones for the whole of the mobility network. Similar to^8^, for the undirected *GC* corresponding to the whole network, the number of pairs of connected and unconnected zones were estimated. Various similarity metrics (local, quasilocal, and global) were calculated between pairs of areas. In particular, the following local similarity indexes were computed: Adamic and Adar^43^, common neighbours, cosine^44^, cosine similarity on L+^45,46^ promoted^47^, jaccard^48^, hub depressed^49,50^, Leicht et al.^51^, preferential attachment^52^, and Sørensen^53^. The global similarity metrics used were: average commute time^54^, normalised average commute time^55^, Katz^56^, L+ directly^46^, matrix forest^57^, and random walk with restart^58^. Finally, the calculated quasilocal similarity metrics were graph distance and local path^50,59^ (see the Supplementary Material Document).

With the purpose of choosing those similarity metrics to be utilised as input variables to the model the existing correlation between them was calculated through the Spearman’s method. This procedure was utilised because according to the results of the Anderson–Darling test, the affinity metrics did not exhibit a normal distribution. Only those metrics with a correlation less than or equal to 0.75 were considered.

The model has the similarity metrics between pairs of nodes as input variables and the indicator of whether a link exists between them as output variable. In order to estimate the model, a cross-validation mechanism was applied where *kf* folds were utilised. The model was trained *kf* times, where each time 1 fold was taken as a test set, and each of the remaining $k-1$ folds were utilised as training set. To know the adequacy of the model, an average of an estimated parameter (*ESTP*) was carried out:

8 $$\begin{aligned} <ESTP>=\frac{1}{kf} \sum _{i=1} ^{i=kf} ESTP_{i} \end{aligned}$$

*ESTP* is Accuracy, Sensitivity, Specificity, Precision, *F*1, *GMean*, *ROC* and kappa. An independent end estimation of the aforementioned performance parameters of the model was also computed using the validation set.

A value *kf* equal to 5 was taken. The dataset used consisted of 53,942 pairs of connected nodes, including those constituting the *GC*, as well as an analogous amount of randomly chosen unconnected pairs taken from all the unconnected pairs existing in the *GC*. 80% of the dataset was taken as training+test set, and 20% as validation set. Performance metrics and the importance of each explanatory variable, computed through the absolute value of the *t*-*statistics* have been included in the Supplementary Material Document.

## Discussion

This paper analyses the mobility of the inhabitants of the Madrid Community, in which various aspects have been examined, through diverse procedures:

(i) We used a non-parametric clustering method which was applied in a more confined environment, considering a larger area as well as the 24 trip typologies. The algorithm generates a $2\times 2$ matrix from the initial *OD* matrix, symbolising the percentage of four major flows: *HH* (origin: hotspot, destination: hotspot), *HN* (origin: hotspot, destination: non-hotspot), *NH* (origin: non-hotspot, destination: hotspot) and *NN* (origin: non-hotspot, destination: non-hotspot). A temporary representation covering the whole day shows that the fluxes were highly linearly related. Maximum values were also detected at the same time. The HH displacement is the one showing the highest number of trips. The main areas with hotspot status were identified in the region. Various commonalities were found between flows, in terms of travel distances and speeds.
(ii) The cumulative probability distributions of trip start times were also analysed by trip type. High similarities were identified between Subway, Urban bus, passenger in private car, Private bicycle and Walking.
(iii) Utilising the *OD* matrix the transition probabilities between areas in the region were also computed and the most likely distances and duration of trips were obtained. As a result, the distances most frequently covered between areas were estimated for each type of transport used. A tendency to move within the same zone or between surrounding sectors has been found. The most probable and largest distances correspond to Long-distance bus, Rest of trains, passenger in company car, and Commuter trains. Similarly to^30^, we obtained an “OD rank” for all types of trip.
(iv) Utilising the Network Theory, an analysis of the mobility networks was performed. Only networks corresponding to trip types: Commuter trains, subway , driver/passenger in private car, and Public and motorbike/moped were fully connected. The mobility networks Walking, Others, Private bicycle, and passenger in company car, show the highest number of disjoint subgraphs (> 100). In addition, some public transport networks, not being fully connected, force the traveller to change means of transport to reach specific areas, even if the destination can be located in the same network.
(v) We prove that link formation characterising mobility between zones using 24 different trip typologies can be correctly reproduced by a *GLM* model. This model also proved to be suitable for anticipating link formation in certain public transport networks^40^.

## Methods

### *OD* matrix construction and characterisation of zones

The OD matrix was generated utilising data collected in the Madrid Community’s Mobility Survey, conducted in 2018 by Consorcio Regional de Transportes de Madrid. In this survey, each trip is described by both origin and destination zones as well as by the main mode of transportation used and by the priority motive for a trip (see Datasets section).

Using the OD matrix it is possible to establish the importance of each zone as both origin and destination of the trip, categorising it as a hotspot or non-hotspot. The above determines the four types of displacements that can be made between zones: *HH*, *HN*, *NH* and *NN*, which can be defined as^11^:

9 $$\begin{aligned} HH= & {} \sum _{i\in M,j\in P}{F}_{ij}/\sum _{i,j}{F}_{ij} \end{aligned}$$

10 $$\begin{aligned} NH= & {} \sum _{i\notin M,j\in P}{F}_{ij}/\sum _{i,j}{F}_{ij} \end{aligned}$$

11 $$\begin{aligned} HN= & {} \sum _{i\in M,j\notin P}{F}_{ij}/\sum _{i,j}{F}_{ij} \end{aligned}$$

12 $$\begin{aligned} NN= & {} \sum _{i\notin M,j\notin P}{F}_{ij}/\sum _{i,j}{F}_{ij} \end{aligned}$$

where *M* and *P* are the origin and destination hotspots, respectively, all trips can be classified into one of the four categories mentioned above. The sum of the trips grouped into each one corresponds to the total number of trips. Similarly to^31^, in order to separate hotspot and non-hotspot zones, we utilised a centroid-based clustering method, which breaks a sorted list of scalars into two categories of higher and lower values. Initially, we sort both the outflow and inflow values of the zones, which correspond to row and column sums of the OD matrix. Analogously to^31^, for n sorted row sums as $qu_{1}> qu_{2}> \cdots > qu_{n}$, we use the clustering algorithm to find the separation point $c_{origin}$ establishing the number of origin hotspots corresponding to the $c_{origin}$ largest outflow magnitudes, i.e. $qu_{1}, qu_{2},\ldots , qu_{c_{origin}}$. For n sorted column sums as $qu_{1}> qu_{2}> \cdots > qu_{n}$, we utilise the clustering algorithm to find the separation point $c_{destination}$ detecting the number of destination hotspots corresponding to the $c_{destination}$ largest inflow magnitudes^31^, i.e. $qu_{1}, qu_{2}, \ldots , qu_{c_{destination}}$. $c_{origin}$ and $c_{destination}$ can be estimated as follows^31^:

13 $$\begin{aligned} \mathop {{\mathrm{\arg }}\,{\mathrm{\min }}}\limits _{c}\,\sum _{i\mathrm {=1}}^{c}\left| {qu}_{i}-\frac{1}{c}\left( \sum _{k\mathrm {=1}}^{c}{qu}_{k}\right) \right| +\sum _{j=c+1}^{n}\left| {qu}_{j}-\frac{1}{n-c}\left( \sum _{l=c+1}^{n}{qu}_{l}\right) \right| \end{aligned}$$

where $q_{i}$ can be either the sum of a row or a column from the *OD* matrix. Resolving Eq. (13) for sorted lists of row sums and column sums, $c_origin$ and $c_destination$ are obtained, respectively^31^.

### Construction of the model describing the interzone links formation

Considering the output $Y_{i}$ and the set of explanatory variables $X_{i}$, ($X_{i1}, X_{is}$) for i = 1, ..., *s*. A *GLM* model incorporating both a random and a systematic element, as well as a link function was constructed. Regarding the random element, it can be accepted that $Y_{i}, 1 \le i \le n$, are independent random variables described by a probability density function belonging to the exponential family:

14 $$\begin{aligned} f(y;\Theta ,\phi ) = exp [(y\Theta - b())/(a(\phi )) + c(y,\phi ))] \end{aligned}$$

where a, b, c symbolise known functions, and $\Theta ,\phi$ represent a natural and a dispersion parameters, respectively.

The systematic element relates some vector ($\eta _1\dotsc \eta _(n)$) to the *s* features.

15 $$\begin{aligned} \eta _i (\beta )=x\beta _{i}^{t}\beta = \beta _{0}+ \beta _{1} x_{i1\Theta }+ \beta _{2} x_{i2}+\cdots +\beta _{s} x_{is} \end{aligned}$$

where $\beta = (\beta _{0},\beta _{1},\ldots ,\beta _{s})$ are the regression parameters. We utilised as link function *g*, a logit function, which returns values in the [0, 1] interval for any input:

16 $$\begin{aligned} g(\mu _{i} )= ln ( \mu _{i}/(1-\mu _{i}) ) \end{aligned}$$

In order to estimate the parameters that correspond to an exponential family *GLM*, the maximum likelihood mechanism was applied.

17 $$\begin{aligned} L(\Theta )= \prod _(i=1)^{n}= f(y_{i};\Theta ,\phi ) \end{aligned}$$

We can compute $\beta$ as in ${\hat{\beta }}$ and then use this estimation to state that

18 $$\begin{aligned} g((\mu _{i}) )= x_{i}^{t} {\hat{\beta }} i=1\ldots n; \hat{\mu _{i}} = g^{-1} (x_(i)^{t}{\hat{\beta }}),i=1\ldots n \end{aligned}.$$

The importance of the predictors, is stated using a *t*-*statistic* estimator, which is defined as:

19 $$\begin{aligned} t\text {-}statistic (\beta _{j}) = \frac{\beta _{j}}{ SE(\beta _{j})} \end{aligned}$$

where $SE(\beta _{j})$ is the standard error of the calculation.

### Software programs

Various functionalities were coded in R language: (1) Network analysis and graph management were performed utilising the igraph package. (2) Modeling was implemented using the caret package. The calculation of the importance of the explanatory variables was made using the vip package. The estimation of similarities between zones was done using the linkprediction package. (3) Maps were built using maps, ggspatial, sf and nortest packages. (4) Plots were made using bothggplot2 and ggplot packages. Finally, the transition probabilities representations were carried out using the hexbin package.

## Supplementary Information

Supplementary Information.

## Data Availability

The main dataset analysed in this study corresponds to the Mobility survey which was conducted in 2018 by the Regional Transport Consortium of Madrid^27,60,61^ (see the Supplementary Material Document). Datasets containing other complementary information related to the existing transport networks in the Madrid Community were also used^34^: (1) elements of the Intercity Bus Network (8515 objects) (2) components of the Urban Bus Network (4,721 objects) (3) parts of the Commuter Network (113 objects) (4) elements of the light subway network (57 elements ), (5) components of the Subway Network (295 elements). Information retrieved from^34,62–64^ was utilised for the construction of maps. Any data not presented in the Manuscript/Supplementary Material Document is available from the corresponding author upon request. Correspondence and requests for materials should be addressed to M.L.M.-L.
