# Supplementary material for: Exploring the mobility in the Madrid Community
Source: Sci Rep. 2023 Jan 17;13:904. doi: 10.1038/s41598-023-27979-5 (PMC9845334; doi:10.1038/s41598-023-27979-5)
Supplement: Supplementary file 1 — Supplementary Information. [file 41598_2023_27979_MOESM1_ESM.pdf]

Exploring the mobility in the Madrid Community. Supplementary Material

Mouronte-López, Mary Luz[1,*] **and Gómez, Javier**[1,2]

[1]Higher Polytechnic School. Universidad Francisco de Vitoria, Carretera Pozuelo a, Av de Majadahonda, Km 1.800, 28223 Madrid, Spain
[2]Grupo de Sistemas Complejos, Escuela Técnica Superior de Ingeniería Agronómica, Alimentaria y de Biosistemas, Universidad Politécnica de Madrid, Avda. Puerta de Hierro 2-4, 28040 Madrid, Spain
[*]maryluz.mouronte@ufv.es

ABSTRACT

Displacements within urban spaces have attracted particular interest among researchers. We examine the journeys that happen in the Madrid Community considering 24 travel typologies and 1,390 administrative areas. From an origin-destination (OD) matrix, four classes of major flows are characterised through coarse-graining: hotspot - non-hotspots, non-hotspot hotspots, hotspots - hotspots, and non-hotspot - non-hotspot. In order to make comparisons between them with respect to spatial and temporal patterns, several statistical tests are performed. The spatial activity as well as transition probabilities between administrative zones are also analysed. The mobility network's topology is examined (some parameters such as maximal connected components, average degree, betweenness, and assortativity as well as the k-cores are checked). A model describing the formation of links between zones (existence of at least one trip between them) is constructed based on certain measures of affinity between areas.

Data Availability, mobility survey, and description of the sample

Since we do not have data on all the inhabitants of the Madrid Community, we utilised a survey as a base for the research performed by the Regional Transport Consortium of the Madrid Community[1],[2],[3]. To ensure the appropriateness of the sample, the Consortium divided the Madrid Community into 1,259 zones with criteria of socio-economic homogeneity, urban planning, and accessibility to transport infrastructures. The average size of the zones was 5,629 people[1],[2],[3].

Two methodologies were used to collect information1: (i) (Methodology A) Face-to-face interview of all members of the household unit. (ii) (Methodology B) Just one member of the household unit was interviewed by telephone[1],[2].

According to[1],[2],[3] Methodology A uses a systematic sampling with proportional allocation and minimum representation by transport area. A total of 13,009 family units were surveyed[1],[2],[3]. Methodology B used non-probabilistic sampling based on age and sex quotas. In this type of interview, 50,412 individuals were surveyed[1]. Proportionality was maintained between both methodologies. The surveys were carried out from Monday to Thursday provided that they were not preceding public holidays. The population under study was the population over the age of 3 years residing in the Madrid Community.

The core of the data collection work consisted of telephone and face-to-face interviews, and the data quality control and validation by the Consortium[1],[2],[3].

Results

Reasons, trip modes and Trip start times

Table 1. Number and percentage of displacements by priority reason.

Priority reason for the displacement	Number	Percentage	Priority reason for the displacement	Number	Percentage
Home	1,220	0.55%	Accompanying another person	20,766	9.32%
**Work**	**57,207**	**25.68%**	Leisure	12,910	5.80%
Work management	4,613	2.07%	**Sport/walking**	**23,546**	**10.57%**
**Study**	**33,400**	**14.99%**	**Personal business**	**27,913**	**12.53%**
**Shopping**	**27,507**	**12.35%**	Other address	2,630	1.18%
Medical	9,553	4.29%	Other	1,479	0.66%

Considering all types of displacements, Figure 1 shows for each i, both k_i^{in} and k_i^{out} the probability distributions. A significant number of zones have a low number of journeys but others also exist showing a high number of trips, as is indicated

by the (80, $2x10^{-4}$) coordinate in the plot.

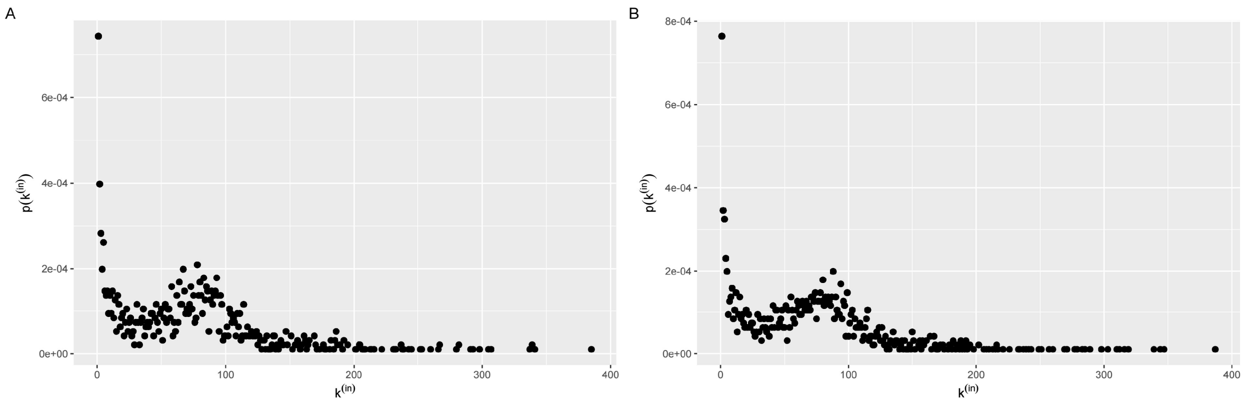

Figure 1. For the whole network, k_i^{in} (Left) and k_i^{out} (Right) probability distributions.

Table 2. Number of times in which $p-value$ is less than 0.05 in the pairwise comparisons, (T_i, T_j), for $j \neq i$, and i, j varying 1, 2, ... 24, between trip start times cumulative probability distributions. (T1): Commuter trains,(T2) Intercity bus, (T3) Urban bus other municipality, (T4) Subway, (T5) Light subway, (T6) Urban bus, (T7) Rest of trains, (T8) Discretionary bus, (T9) Long-distance bus, (T10) Taxi, (T11) Driver in private car, (T12) Driver in company car, (T13) Driver or passenger in rented car without driver, (T14) Passenger in private car, (T15) Passenger in company car, (T16) Passenger in rented car with driver, (T17) Private motorbike, (T18) Public motorbike, (T19) Company motorbike (T20) Private bicycle, (T21) Public bicycle, (T22) Company bicycle, (T23) Other, (T24) Walking.

**Type T1**	**Type T2**	**Type T3**	**Type T4**	**Type T5**	**Type T6**	**Type T7**	**Type T8**	**Type T9**	**Type T10**	**Type T11**	**Type T12**	
7	5	9	6	1	7	6	**18**	0	7	9		
**Type T13**	**Type T14**	**Type T15**	**Type T16**	**Type T17**	**Type T18**	**Type T19**	**Type T20**	**Type T21**	**Type T22**	**Type T23**	**Type T24**	
**10**	1	8	**12**	2	2	6	**16**	4	0	0	8	6

Characterization of the major flows

Table 3. Maximum values of fluxes.

	Time	Number of trips	Time	Number of trips	Time	Number of trips
HH	08:00	13,590	14:00	9,501	17:00	9,574
HN	08:00	4,148	14:00	2,917	17:00	2,981
NH	08:00	4,023	14:00	2,777	17:00	3,140
NN	08:00	2,723	14:00	1,905	17:00	2,021

Table 4. Spearman's correlation coefficient between types of flow.

	HH	HN	NH	NN
**HH**	1.00	0.94	0.96	0.99
**HN**	0.94	1.00	0.97	0.95
**NH**	0.96	0.97	1.00	0.97
**NN**	0.99	0.95	0.97	1.00

Table 5. The top 5 zones with the highest number of trips (both for origin and destination). Shape length and Shape area are respectively expressed in meters and square meters, respectively..

ID	Name	Shape length	Shape area
079-04-075	Salamanca, Madrid	308.538.151.286.079	422.009.981.659.181
079-05-096	El Viso, Chamartín, Madrid	564.065.266.683.902	164.461.404.327.636
005-020	Alcalá de Henares Center	466.707.400.975.314	127.413.065.011.752
079-01-014	Sol, Madrid Center	248.998.269.305.996	275.975.023.016.713
079-06-113	Tetuán, Madrid	379.918.743.626.212	627.343.720.547.598

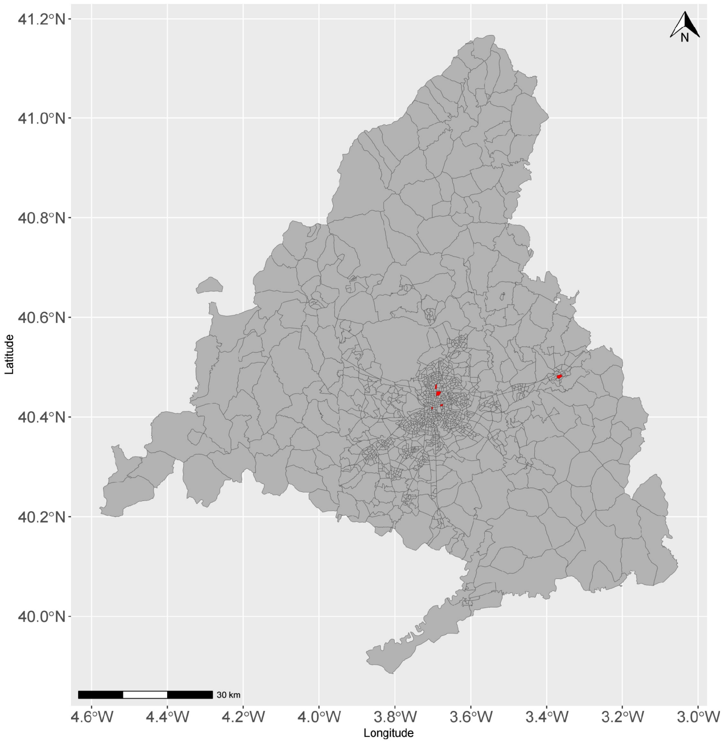

Figure 2. Map showing (in red) the 5 areas with the highest number of trips (both for origin and destination. Information retrieved from[4] has been used for the construction of the map).

Table 6. For the comparison of the distance cumulative probability distributions, the obtained $p - value$ in the Kolmogorov–Smirnov test.

	**HH**	**HN**	**NH**	**NN**
**HH**	1	0	0	0
**HN**	0	1	**0.27**	0
**NH**	0	**0.27**	1	0
**NN**	0	0	0	1

Table 7. For the comparison of the velocity cumulative probability distributions, the obtained $p - value$.

	**HH**	**HN**	**NH**	**NN**
**HH**	1	**0.18**	**0.18**	0.04
**HN**	**0.18**	1	0.01	**0.19**
**NH**	**0.18**	0.01	1	**0.20**
**NN**	0.04	**0.19**	**0.20**	1

Population, land occupation, and number of hotspots

It is relevant for each municipality in the Madrid Community to relate its population and percentage of land occupation to the ratio of hotspots. To achieve a better understanding of this relationship we describe below how land is categorised in Spain.

In Spain land is classified as urban, developable, and non-developable. Urban land can be either consolidated or unconsolidated. Both categories have all the services (such as urbanized streets, lighting, etc), differing only in whether they are occupied. A consolidated land is occupied by a use, while an unconsolidated land is pending occupation. The developable land (not urban yet) is categorised as unbounded or bounded land.

In bounded land urban planning expressly binds as preferential land for urbanizing, guaranteeing a rational urban development. Unbounded land lacks the characteristic of bounded urban land and the urban planning classifies it a developable land. The developable land is programmed to become urban land in the future. Building is forbidden on non-developable land since it is a protected area.

Figures 3 (D), and 4 (A, B, C, D, E) show the percentage of the different types of land, which was calculated over the full area of each municipality. It must be noted that we used the latest information provided by the Ministry of Transport, Mobility and Urban Agenda[5]. The most recent update date varies for each municipality between 2005 and 2022.

The Madrid Community has 179 municipalities. Figure 3 (A) represents the population by municipality in the year 2018[6]. It can be observed that the municipality with the highest population corresponds to Madrid (3,223,334 inhabitants), which is marked in purple colour. The remaining municipalities have a population between 47 and 207,095 inhabitants. Figure 3 (B) depicts the number of hotspots by municipality. Madrid is the municipality with the highest number of hotspots (269). The rest of the municipalities with a considerable number of hotspots are located around the capital city. Figure 3 (C) displays the hotspot - non-hotspot zones by municipality. Figure 3 (D) depicts the rate of consolidated land. 16 municipalities present a percentage higher than 25%. The percentage of this land type in Madrid is 37.78%.

Figure 4 (A, B, C, D, E) represents the ratio of different land types by municipality in the Madrid Community. Figure 4 (A) displays the percentage of developmental areas (unconsolidated + bounded land). 5 municipalities show a rate of more than 25%. The percentage equals 21.79% in Madrid. These magnitudes of development zones could impact mobility in the coming years. Figure 4 (B) shows the rate of unconsolidated land. The magnitudes for all municipalities are lower than 10.10% for all municipalities. The Madrid municipality shows a percentage equal to 8.25%. Figure 4 (C) shows the ratio of bounded land. 5 municipalities have a percentage higher than 25%. The Madrid municipality has a value equal to 13.55%.

Figure 4 (D) depicts the percentage of unbounded land. 10 municipalities exhibit a percentage of more than 25% . The percentage of this land type in the Madrid municipality is equal to 2.20%. Figure 4 (E) presents the rate of of non-developable land. 94 municipalities over the total of 179 present a ratio of more than 90%. The Madrid municipality exhibits a percentage equal to 36.92%

Figure 5 shows the relationships between population and number of hotspots with consolidated land rate. Madrid municipality has been excluded because it has a much higher population and the largest number of hotspots. It is not observed that a higher percentage of consolidated land generally implies a greater number of hotspots in the municipality.

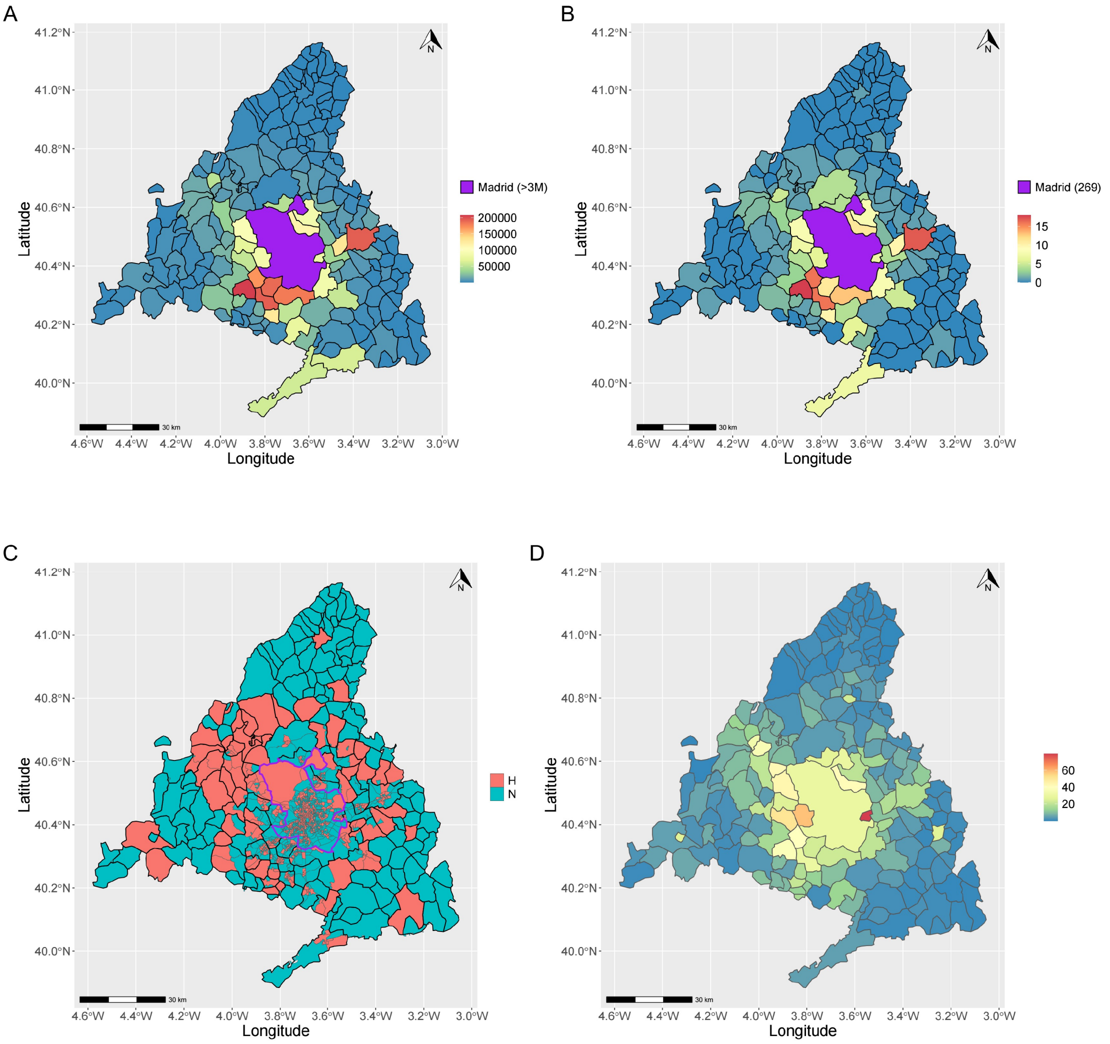

Figure 3. For the Madrid Community are represented: (A) Population, (B) Number of hotspots, (C) Hotspot -non-hotspot zones and (D) Percentage of consolidated land by municipality (Madrid Community). Information retrieved from[7],[8],[9] has been used for the construction of the maps.

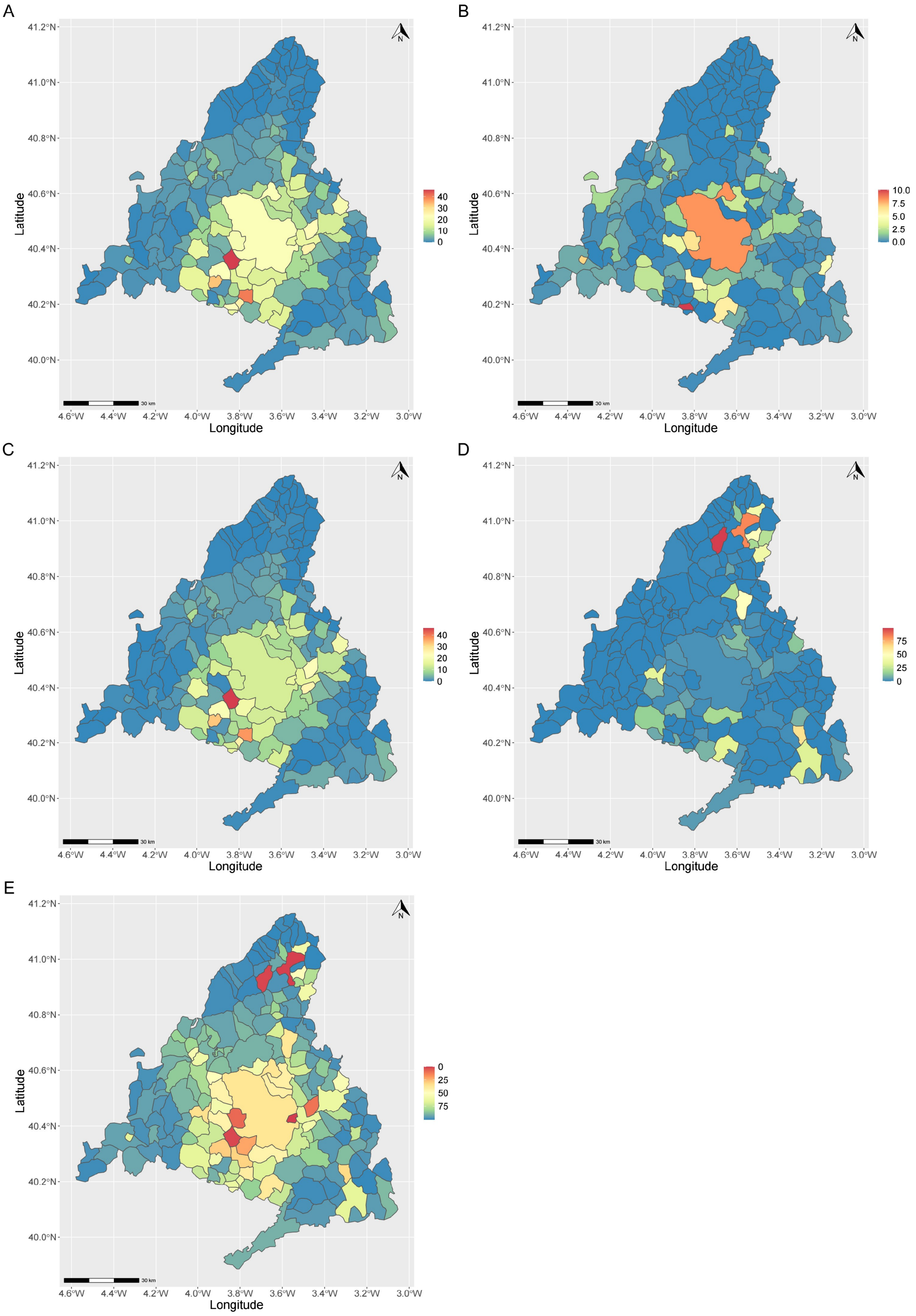

Figure 4. For the Madrid Community are represented: (A) Percentage of developmental areas (unconsolidated + bounded land), (B) Rate of unconsolidated land, (C) Ratio of bounded land, (D) Percentage of unbounded land, and (E) Rate of non developable land (Madrid Community). Information retrieved from[7],[8],[9] has been used for the construction of the maps.

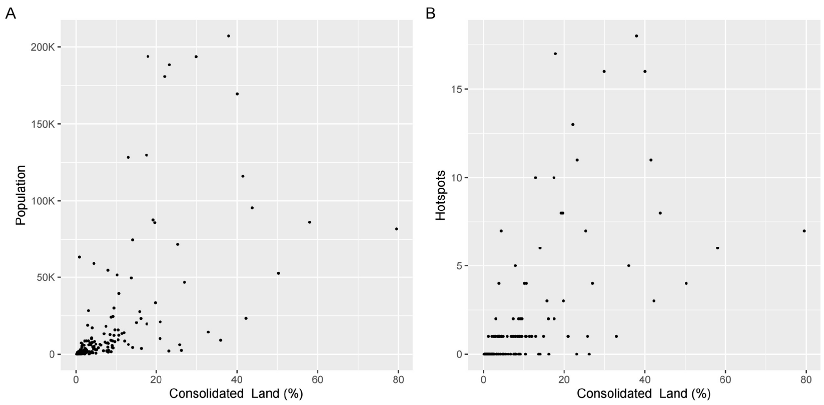

Figure 5. For the Madrid Community are represented: (A) Population and (B) Hotspots as a function of consolidated land ratio (Madrid Community, Madrid municipality has been excluded).

Trip start times.

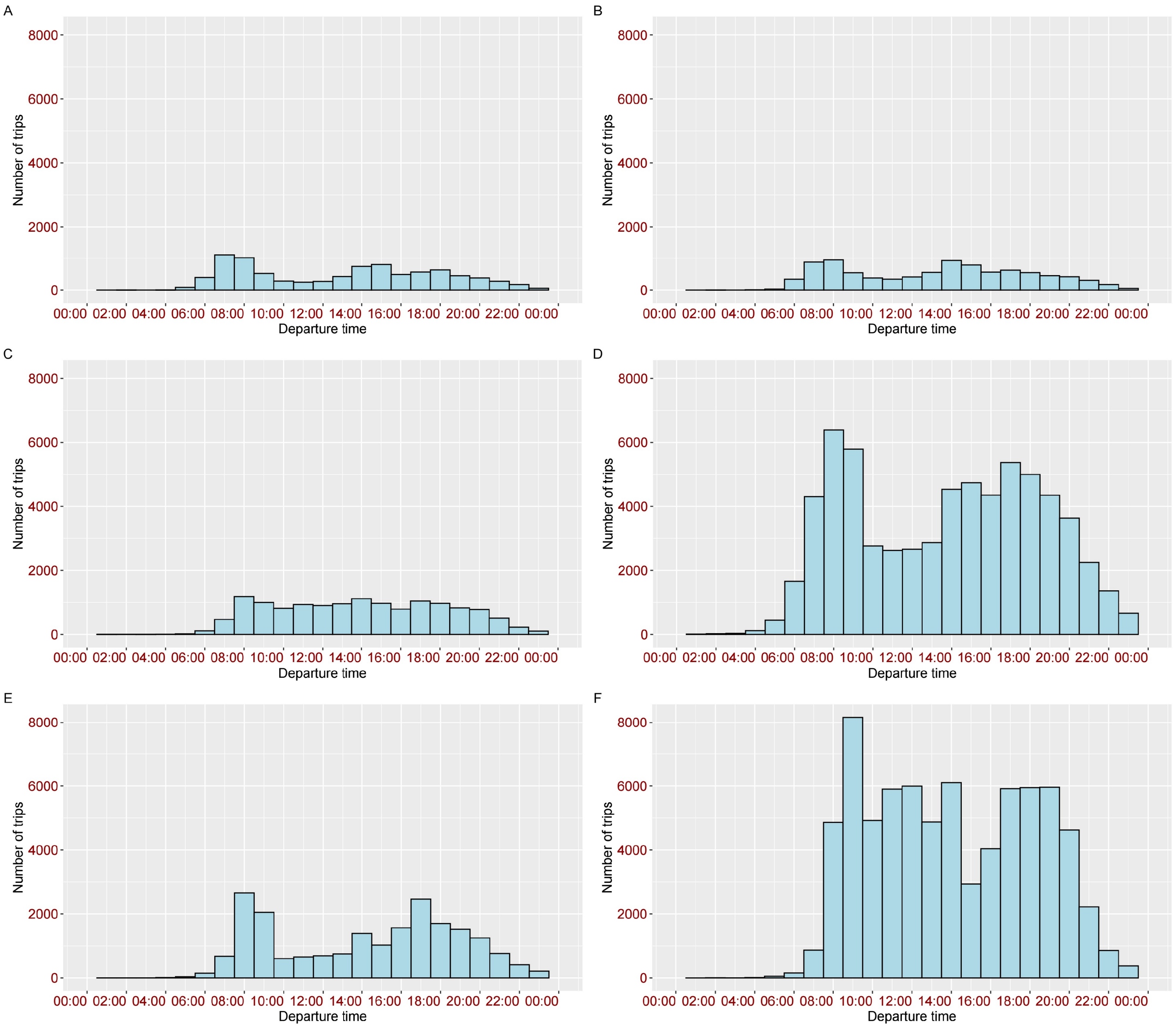

Figure 6. For the trip modes with a usage rate higher than 4%, histogram of trip start time. A: (T1) Commuter trains, B: (T2) Intercity bus, C: (T6) Urban bus, D: (T11) Driver in private car, E: (T14) Passenger in private car, F: (T24) Walking

.

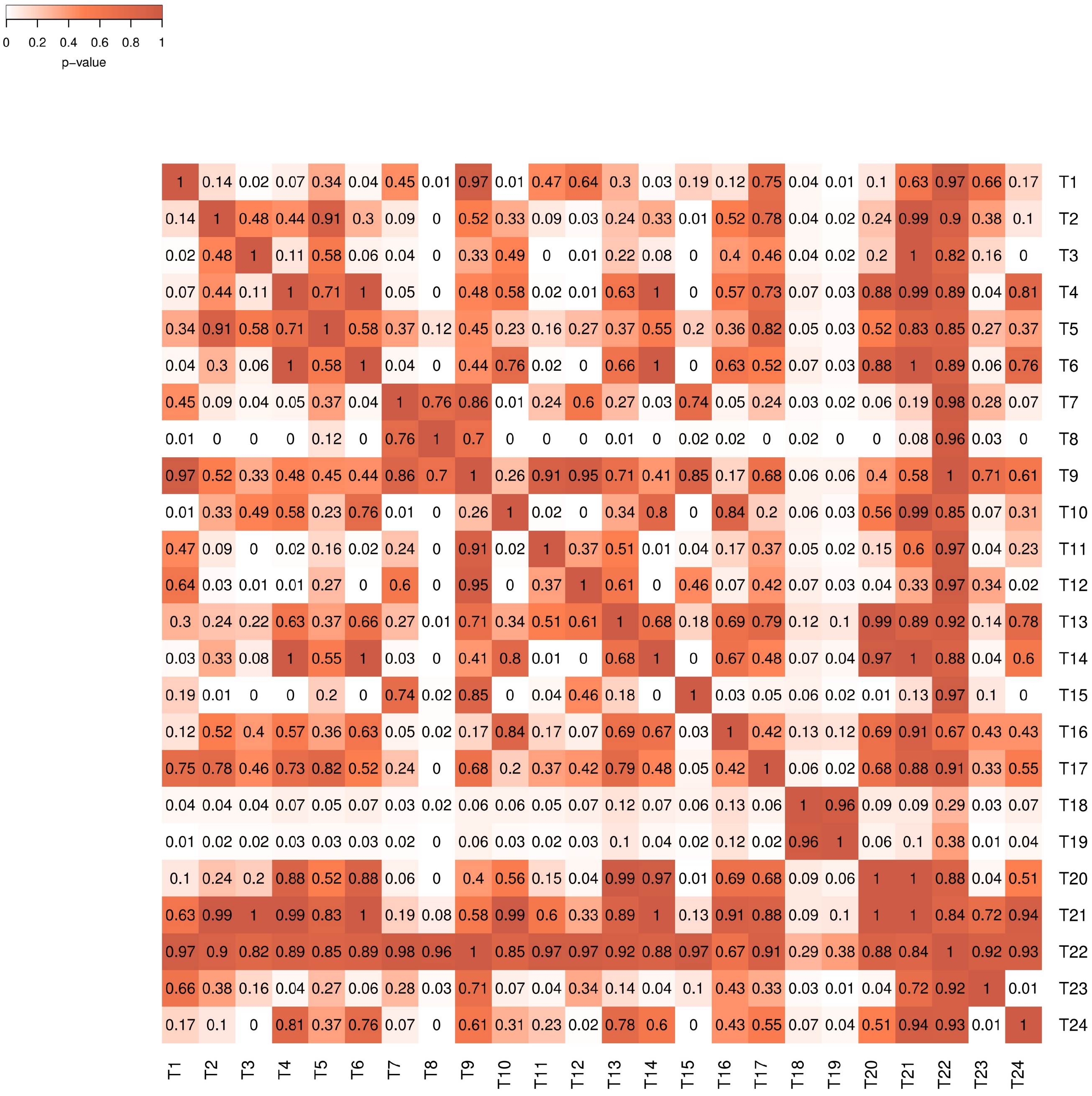

Figure 7. Heatmap showing the obtained p-values in the comparisons between trip start times cumulative probability distributions for all travel types.

Trip distances

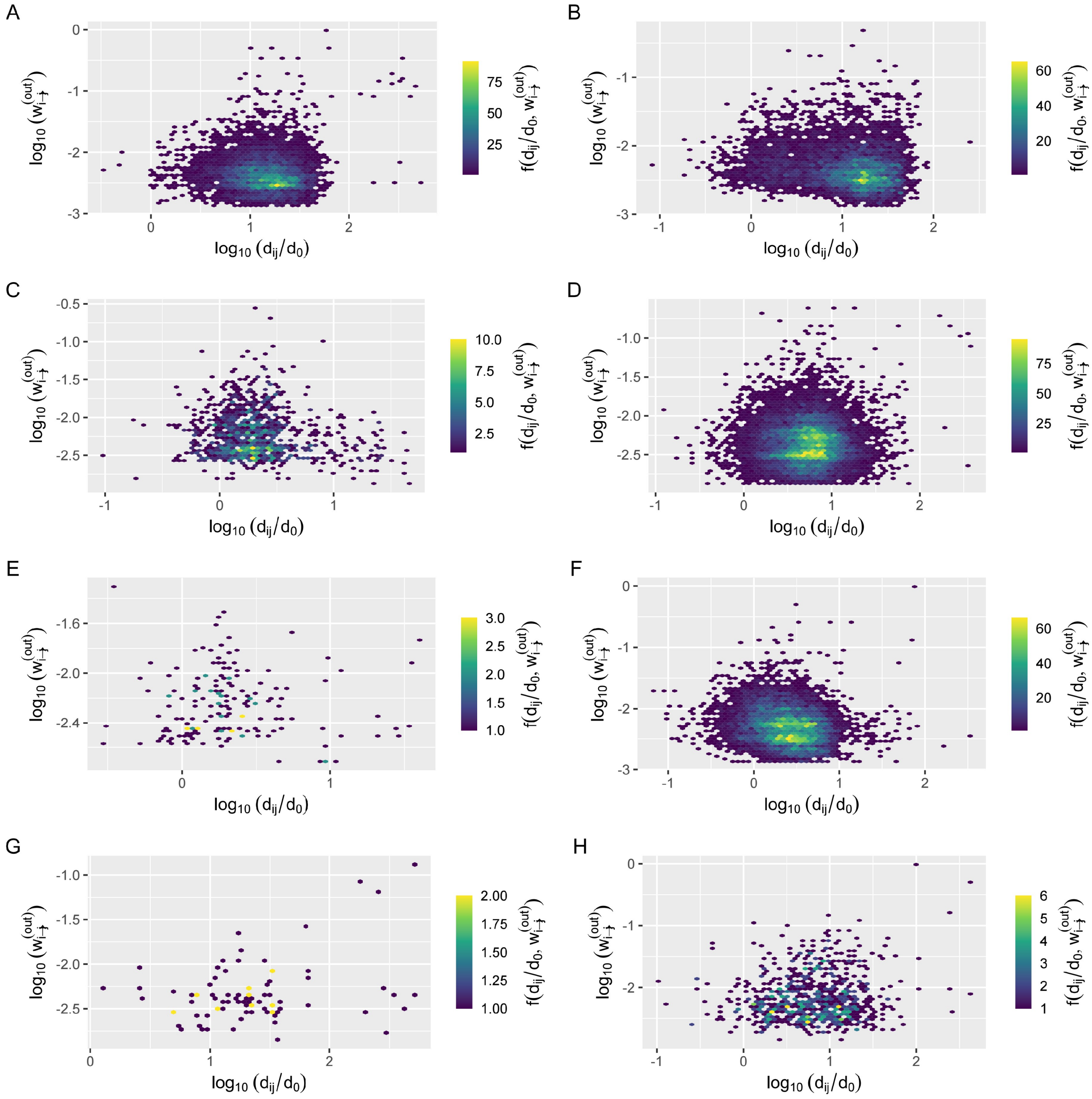

Figure 8. Distances according transition probabilities between zones by trip type A: (T1): Commuter trains,B: (T2) Intercity bus, C: (T3) Urban bus other municipality, D: (T4) Subway, E: (T5) Light subway, F: (T6) Urban bus, G: (T7) Rest of trains, H: (T8) Discretionary bus.

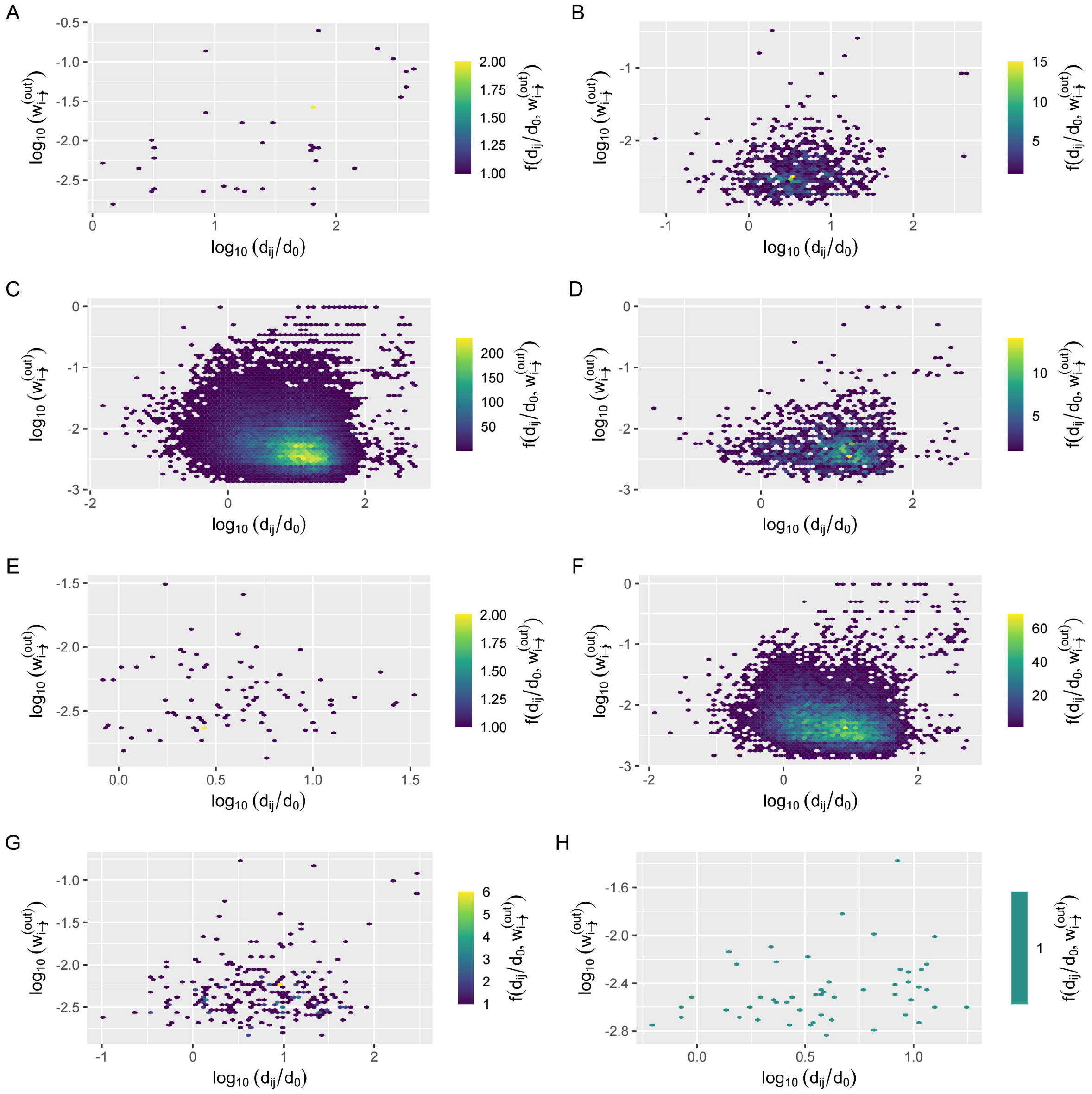

Figure 9. Distances according transition probabilities between zones by trip type A: (T9) Long-distance bus, B: (T10) Taxi, C: (T11) Driver in private car, D: (T12) Driver in company car, E: (T13) Driver or passenger in rented car without driver, F: (T14) Passenger in private car, G: (T15) Passenger in company car, H: (T16) Passenger in rented car with driver.

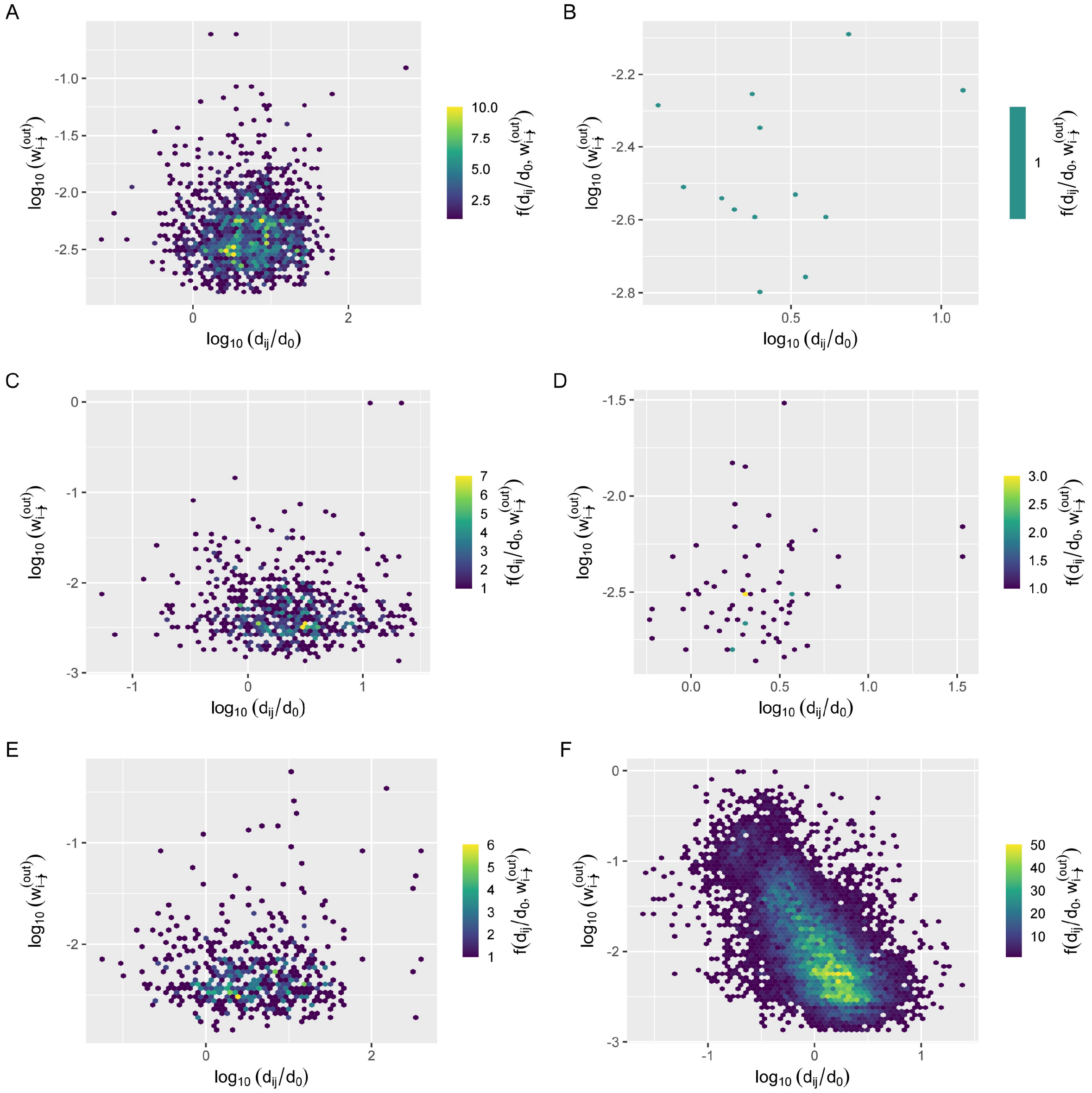

Figure 10. Distances according transition probabilities between zones by trip type A: (T17) Private motorbike, B: (T19) Company motorbike, C: (T20) Private bicycle, D: (T21) Public bicycle, ,E: (T23) Other, F: (T24) Walking. Graphical representations corresponding to (T18) Public motorbike and (T22) Company bicycles are not shown because they exhibited a very small number of trips.

Table 8. Distances (calculated as $log_{10}(\frac{d_{ij}}{d_0})$) corresponding to the top 5 highest transition probabilities between zones by trip type (calculated as $log_{10}w_{i\to j}^{(out)}$). If these top 5 highest transition probabilities are very similar, only one value is shown (T1): Commuter trains,(T2) Intercity bus, (T3) Urban bus other municipality, (T4) Subway, (T5) Light subway, (T6) Urban bus, (T7) Rest of trains, (T8) Discretionary bus, (T9) Long-distance bus, (T10) Taxi, (T11) Driver in private car, (T12) Driver in company car, (T13) Driver or passenger in rented car without driver, (T14) Passenger in private car, (T15) Passenger in company car, (T16) Passenger in rented car with driver, (T17) Private motorbike, (T18) Public motorbike, (T19) Company motorbike (T20) Private bicycle, (T21) Public bicycle, (T22) Company bicycle, (T23) Other, (T24) Walking.

Trip type	Distance	Transition probability	Number of trips	Trip type	Distance	Transition probability	Number of trips
T1	1.2693089	-2.537403	91	T11	0.9843196	-2.288971	223
T1	1.3221969	-2.537403	76	T11	0.9843196	-2.454592	217
T1	1.2164210	-2.537403	74	T11	1.2493290	-2.495998	213
T1	1.1106452	-2.454592	65	T12	T1.16568551	-2.454592	14
T1	1.3750848	-2.537403	63	T12	1.09711572	-2.371782	11
T2	1.19948573	-2.502300	65	T12	1.06283082	-2.330376	11
T2	1.25799728	-2.502300	56	T12	1.19997041	-2.247566	11
T2	1.22874151	-2.539361	55	T12	1.23425531	-2.537403	10
T2	1.22874151	-2.391120	55	T13	0.44032656	-2.630220	2
T2	1.25799728	-2.428180	51	T14	0.91916920	-2.371782	68
T3	0.28789728	-2.536645	10	T14	1.07236423	-2.537403	54
T3	0.28789728	-2.402436	10	T14	0.88087044	-2.495998	54
T3	0.17716717	-2.435988	9	T14	1.14896175	-2.537403	53
T3	0.28789728	-2.469541	8	T14	0.91916920	-2.454592	53
T3	0.44291943	-2.435988	8	T15	0.95867498	-2.2327674	6
T4	0.8782933	-2.478424	95	T15	1.39443678	-2.5609393	3
T4	0.6733580	-2.511139	93	T15	0.98772576	-2.5012717	3
T4	0.7026344	-2.478424	93	T15	0.92962419	-2.4416041	3
T4	0.7611874	-2.478424	92	T15	0.14525294	-2.4117703	3
T4	0.8490168	-2.511139	89	T16	0.59768355	-2.835386	1
T5	0.33436158	-2.468653	3	T17	0.52119253	-2.543855	10
T5	0.03519506	-2.448241	3	T17	0.42346874	-2.511139	10
T5	0.10558718	-2.448241	3	T17	0.52119253	-2.478424	10
T5	0.40475370	-2.346185	3	T17	0.87951308	-2.249417	10
T5	0.96789068	-2.713589	2	T17	0.94466227	-2.445709	9
T6	0.40039969	-2.495998	66	T18	-0.3583334	-2.660416	1
T6	0.43119974	-2.454592	63	T19	0.39681951	-2.797449	1
T6	0.30799953	-2.288971	61	T20	0.47496346	-2.495998	7
T6	0.61600006	-2.288971	60	T20	0.49758082	-2.454592	7
T6	0.43119974	-2.537403	59	T20	0.09046842	-2.454592	6
T7	0.6943214	-2.538751	2	T20	0.52019817	-2.495998	5
T7	1.5188292	-2.538751	2	T20	0.61066760	-2.495998	5
T7	1.0631802	-2.500227	2	T21	0.30630046	-2.510018	3
T7	1.3452486	-2.461704	2	T21	0.23337154	-2.802330	2
T7	1.5188292	-2.461704	2	T21	0.30630046	-2.665917	2
T8	0.74566306	-2.557641	6	T21	0.56884456	-2.510018	2
T8	0.32809119	-2.393379	6	T21	0.36464359	-2.860792	1
T8	0.50705056	-2.311248	6	T22	0.4946319	-2.230477	1
T8	1.10358181	-2.311248	6	T23	0.38457941	-2.516575	6
T8	1.13340837	-2.516575	5	T23	0.28843431	-2.475510	5
T9	1.8130660	-1.5735309	2	T23	1.18578860	-2.393379	5
T10	0.53550426	-2.490124	15	T23	0.83325656	-2.270183	5
T10	0.50400395	-2.524642	12	T23	0.03204737	-2.475510	4
T10	0.53550426	-2.559161	8	T24	0.22263250	-2.247566	50
T10	0.59850488	-2.559161	8	T24	0.32158073	-2.247566	49
T10	0.37800271	-2.524642	8	T24	0.22263250	-2.330376	48
T11	1.0978951	-2.495998	231	T24	0.12368428	-2.247566	47
T11	1.0600366	-2.454592	227	T24	0.17315839	-2.247566	47

Table 9. Statistical quartiles, minimum and maximum of travel distance by trip type. Distance is expressed in kilometers (T1): Commuter trains,(T2) Intercity bus, (T3) Urban bus other municipality, (T4) Subway, (T5) Light subway, (T6) Urban bus, (T7) Rest of trains, (T8) Discretionary bus, (T9) Long-distance bus, (T10) Taxi, (T11) Driver in private car, (T12) Driver in company car, (T13) Driver or passenger in rented car without driver, (T14) Passenger in private car, (T15) Passenger in company car, (T16) Passenger in rented car with driver, (T17) Private motorbike, (T18) Public motorbike, (T19) Company motorbike (T20) Private bicycle, (T21) Public bicycle, (T22) Company bicycle, (T23) Other, (T24) Walking.

Trip type	**Quartil 1**	**Quartil 2**	**Quartil 3**	**Minimum**	**Maximum**	Trip type	**Quartil 1**	**Quartil 2**	**Quartil 3**	**Minimum**	**Maximum**
All	0.70392	2.22049	7.81972	0.00385	527.54004	T13	2.31070	3.60718	6.10942	0.83420	33.30122
**T1**	**9.01454**	**14.32909**	**21.24653**	**0.34811**	**518.78824**	T14	1.28805	3.17087	9.02042	0.01298	511.89429
**T2**	**4.66483**	**12.70953**	**21.72223**	**0.08128**	**300.85654**	T15	2.24105	5.80170	13.47458	0.10277	314.73956
**T3**	**1.25438**	**1.81153**	**2.64839**	**0.09662**	**43.91349**	T16	2.29355	3.85896	8.35043	0.61297	17.83012
T4	3.28443	5.26909	7.95433	0.11582	377.54667	T17	2.42971	4.92089	9.44800	0.06344	514.44259
T5	1.22640	1.84573	2.45116	0.30697	39.70623	**T18**	**0.43264**	**0.67792**	**3.41596**	**0.37841**	**3.41596**
T6	1.37312	2.27870	3.75380	0.06270	355.59820	T19	2.03811	2.50853	3.50760	1.15279	11.88256
**T7**	**11.22383**	**20.61190**	**32.87156**	**1.26577**	**508.23527**	T20	0.89006	1.88539	3.42388	0.05340	27.64451
T8	2.87798	6.05053	11.83448	0.10726	407.04710	T21	1.66642	2.14683	3.30518	0.58478	32.90670
**T9**	**3.35381**	**24.05075**	**68.50851**	**1.21535**	**411.68183**	T22	1.72772	2.19598	2.66423	1.25946	3.13249
T10	2.16933	3.76993	7.46447	0.06923	417.21693	T23	1.09738	2.91986	8.44678	0.05840	409.47614
T11	1.89039	5.70084	13.33333	0.01333	527.54004	**T24**	**0.26597**	**0.50934**	**0.90568**	**0.00385**	**16.60512**
T12	3.82538	10.29962	19.54220	0.03954	506.22000						

Similarity between travel distances

A Manhattan distances matrix considering the median trip distances between zones is calculated. This will allow us to assess the similarity between median trip distances from each origin to all destinations.

For the analysed trips there are: 1,384 origin and 1,372 destination zones (1,390 different zones) for the analysed trips. A vector $\overrightarrow{MD_i} = (md_{i1}, \ldots, md_{i1,372})$ can be built, where i varies from 1 to $1,384$. 1,384 vectors $\overrightarrow{MD_i}$ exist whose coordinates are the median distances (in kilometers) for all trips from zone i to zone j where j varies from 1 to 1,372. A Manhattan distance matrix is estimated as a similarity metric between the aforementioned vectors. It must be noted that although 1,259 zones exist in the Madrid Community, a few trips originate in or are destined for other communities.

The distances have been normalised with respect to their maximum value. The lower the Manhattan distance, the more similar the vectors are. Figure 11 shows the frequency distribution of the calculated normalised Manhattan distances. It can be observed that a high similarity exists between many pairs of vectors.

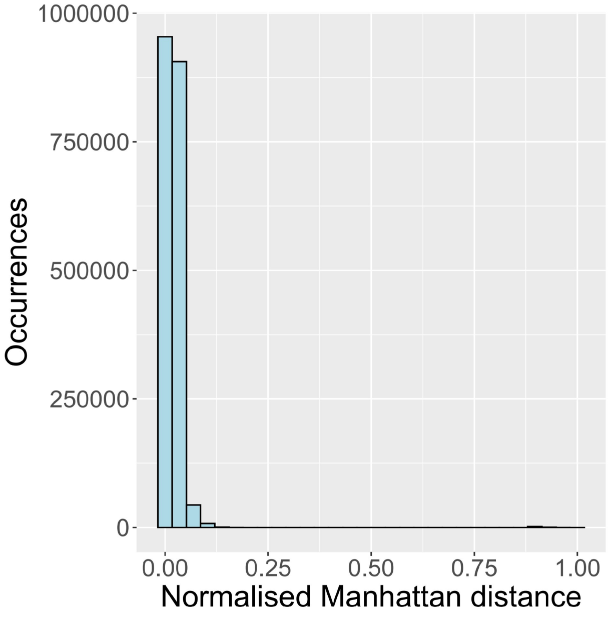

Figure 11. Histogram of the normalised Manhattan distances of the vectors symbolising the mediam distances for all trips from one origin. The Manhattan distances have been normalised with respect their highest value.

Trip durations

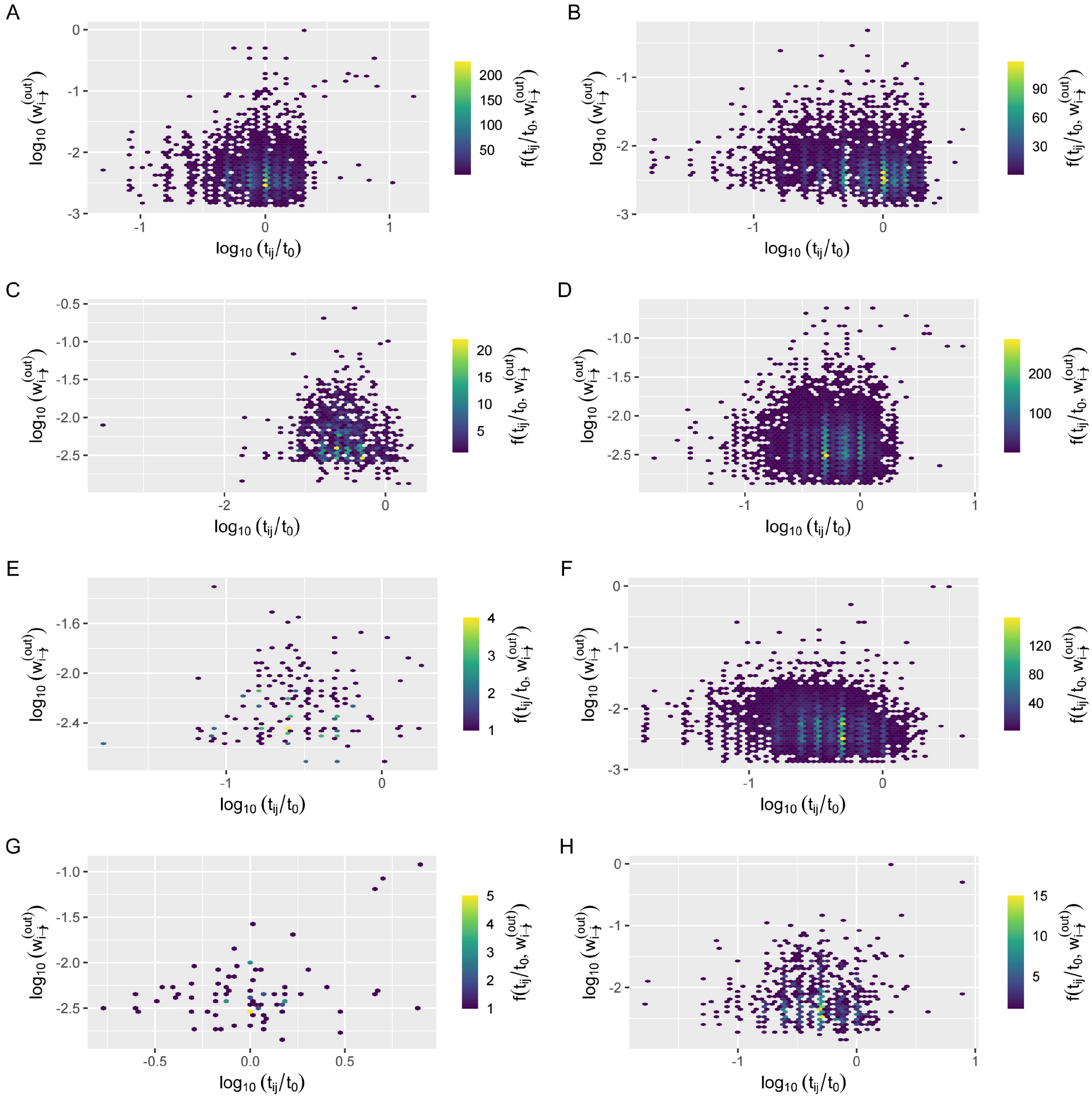

Figure 12. Time traveling according transition probabilities between zones by trip type A: (T1): Commuter trains, B: (T2) Intercity bus, C: (T3) Urban bus other municipality, D: (T4) Subway, E: (T5) Light subway, F: (T6) Urban bus, G: (T7) Rest of trains, H: (T8) Discretionary bus.

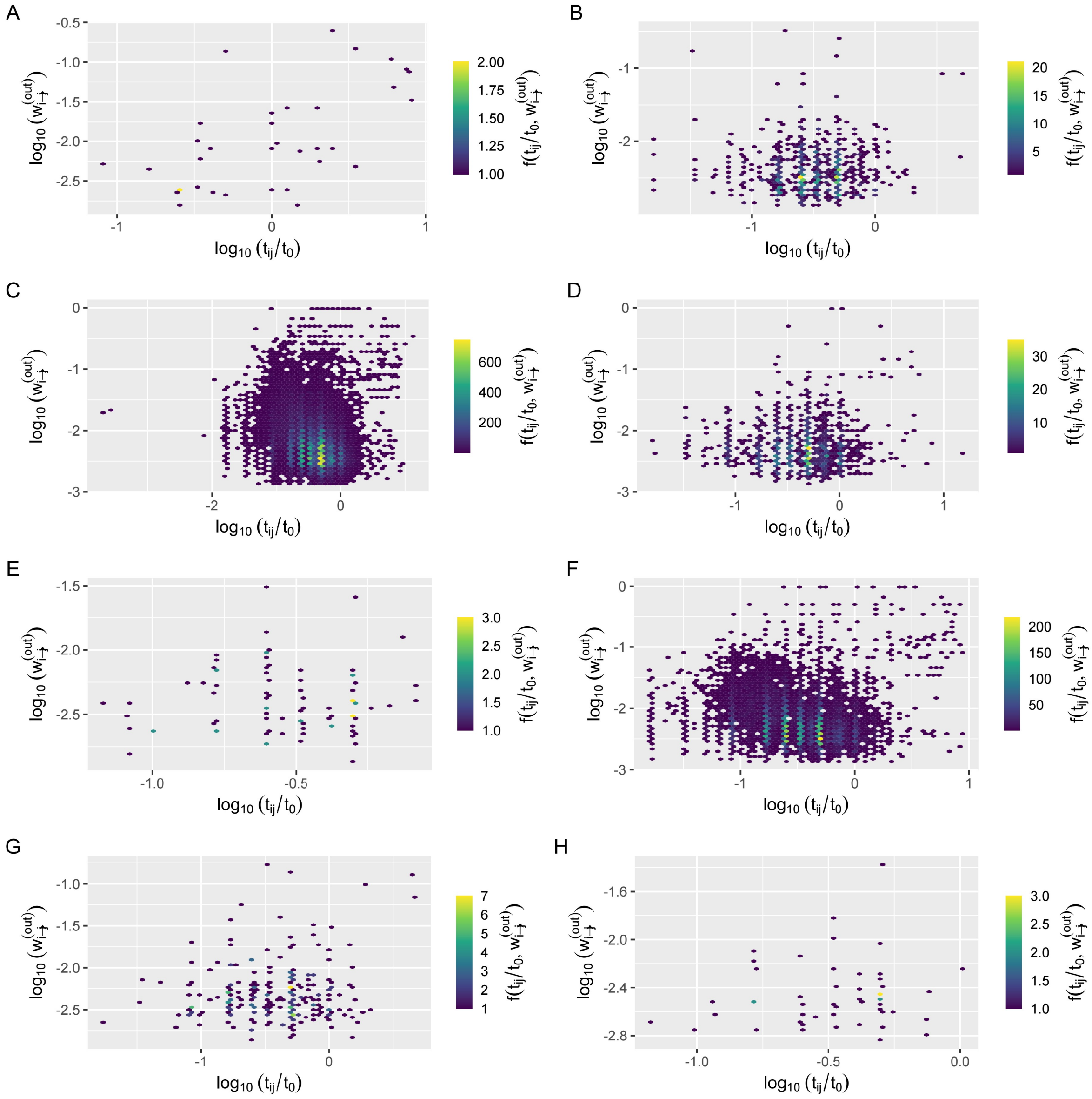

Figure 13. Time traveling according transition probabilities between zones by trip type A: (T9) Long-distance bus, B: (T10) Taxi, C: (T11) Driver in private car, D: (T12) Driver in company car, E: (T13) Driver or passenger in rented car without driver, F: (T14) Passenger in private car, G: (T15) Passenger in company car, H: (T16) Passenger in rented car with driver.

Table 10. Statistical quartiles, minimum and maximum time of travel duration by trip type. Duration is expressed in minutes. (T1): Commuter trains,(T2) Intercity bus, (T3) Urban bus other municipality, (T4) Subway, (T5) Light subway, (T6) Urban bus, (T7) Rest of trains, (T8) Discretionary bus, (T9) Long-distance bus, (T10) Taxi, (T11) Driver in private car, (T12) Driver in company car, (T13) Driver or passenger in rented car without driver, (T14) Passenger in private car, (T15) Passenger in company car, (T16) Passenger in rented car with driver, (T17) Private motorbike, (T18) Public motorbike, (T19) Company motorbike (T20) Private bicycle, (T21) Public bicycle, (T22) Company bicycle, (T23) Other, (T24) Walking.

Trip type	Quartil 1	Quartil 2	Quartil 3	Minimum	Maximum	Trip type	Quartil1	Quartil 2	Quartil 3	Minimum	Maximum
All	10	20	30	1	975	T13	15	17	30	4	50
**T1**	**40**	**60**	**75**	**3**	**975**	**T14**	**10**	**15**	**30**	**1**	**540**
T2	30	45	70	1	265	T15	10	25	35	1	270
**T3**	**10**	**15**	**30**	**1**	**120**	T16	15	20	30	4	60
T4	25	30	50	1	480	T17	15	20	30	1	300
**T5**	**10**	**15**	**25**	**1**	**105**	**T18**	**6**	**10**	**15**	**5**	**20**
T6	15	20	30	1	240	**T19**	**10**	**15**	**20**	**10**	**30**
**T7**	**43.5**	**60**	**82.5**	**10**	**480**	T20	10	20	30	1	120
T8	15	30	40	1	470	T21	15	20	30	5	180
**T9**	**20**	**60**	**120**	**5**	**480**	**T22**	**45**	**70**	**95**	**20**	**120**
T10	15	20	30	1	320	T23	10	20	30	1	645
T11	10	20	30	1	780	**T24**	**8**	**15**	**20**	**1**	**210**
T12	15	30	45	1	900						

Table 11. Statistical quartiles, minimum and maximum of travel velocity by trip type. Velocity is expressed as kilometers per hour. (T1): Commuter trains,(T2) Intercity bus, (T3) Urban bus other municipality, (T4) Subway, (T5) Light subway, (T6) Urban bus, (T7) Rest of trains, (T8) Discretionary bus, (T9) Long-distance bus, (T10) Taxi, (T11) Driver in private car, (T12) Driver in company car, (T13) Driver or passenger in rented car without driver, (T14) Passenger in private car, (T15) Passenger in company car, (T16) Passenger in rented car with driver, (T17) Private motorbike, (T18) Public motorbike, (T19) Company motorbike (T20) Private bicycle, (T21) Public bicycle, (T22) Company bicycle, (T23) Other, (T24) Walking.

Trip type	Quartil 1	Quartil 2	Quartil 3	Minimum	Maximum	Trip type	Quartil 1	Quartil 2	Quartil 3	Minimum	Maximum
All	3.41796	7.59489	17.11859	0.01196	119.20484	T13	8.83680	12.28032	21.31843	3.17963	66.60244
T1	11.10128	15.59245	21.20145	0.83616	116.30869	T14	7.54602	13.11070	25.53486	0.04197	118.35474
T2	8.74718	14.92941	22.50489	0.75672	93.99378	T15	8.52383	16.24238	28.57414	3.17710	86.94330
**T3**	**4.72626**	**6.53996**	**8.80616**	**1.95442**	**59.00944**	T16	7.65156	12.27730	18.78345	3.42499	58.21714
T4	6.61589	8.85479	12.10912	0.43398	113.53436	T17	10.29965	16.62022	26.71597	3.06771	102.88852
T5	4.97402	6.97758	9.03783	3.02485	67.20347	**T18**	**4.06753**	**5.19164**	**10.24787**	**3.78407**	**13.66382**
**T6**	**4.78648**	**6.48856**	**8.36652**	**0.12541**	**108.05648**	T19	7.42909	12.22869	15.01082	3.75446	47.53023
T7	14.39995	20.98044	32.14746	3.61649	112.40738	T20	3.84347	6.73487	10.38286	0.12730	14.97385
T8	7.81684	13.62824	22.56477	1.29774	118.46649	T21	5.05409	7.20933	9.84617	1.16955	14.62828
**T9**	**14.52260**	**32.74628**	**46.56232**	**6.07452**	**62.60069**	**T22**	**2.82167**	**5.01360**	**7.20554**	**0.62973**	**9.39748**
T10	7.64999	11.83408	19.94504	3.01694	119.20484	T23	6.19170	10.20658	17.91091	0.47979	117.58710
**T11**	**9.52769**	**17.56012**	**30.69632**	**0.05126**	**118.35474**	**T24**	**1.54237**	**2.45959**	**3.55016**	**0.01196**	**42.85670**
**T12**	**12.09189**	**21.76783**	**36.34463**	**0.16325**	**108.73241**						

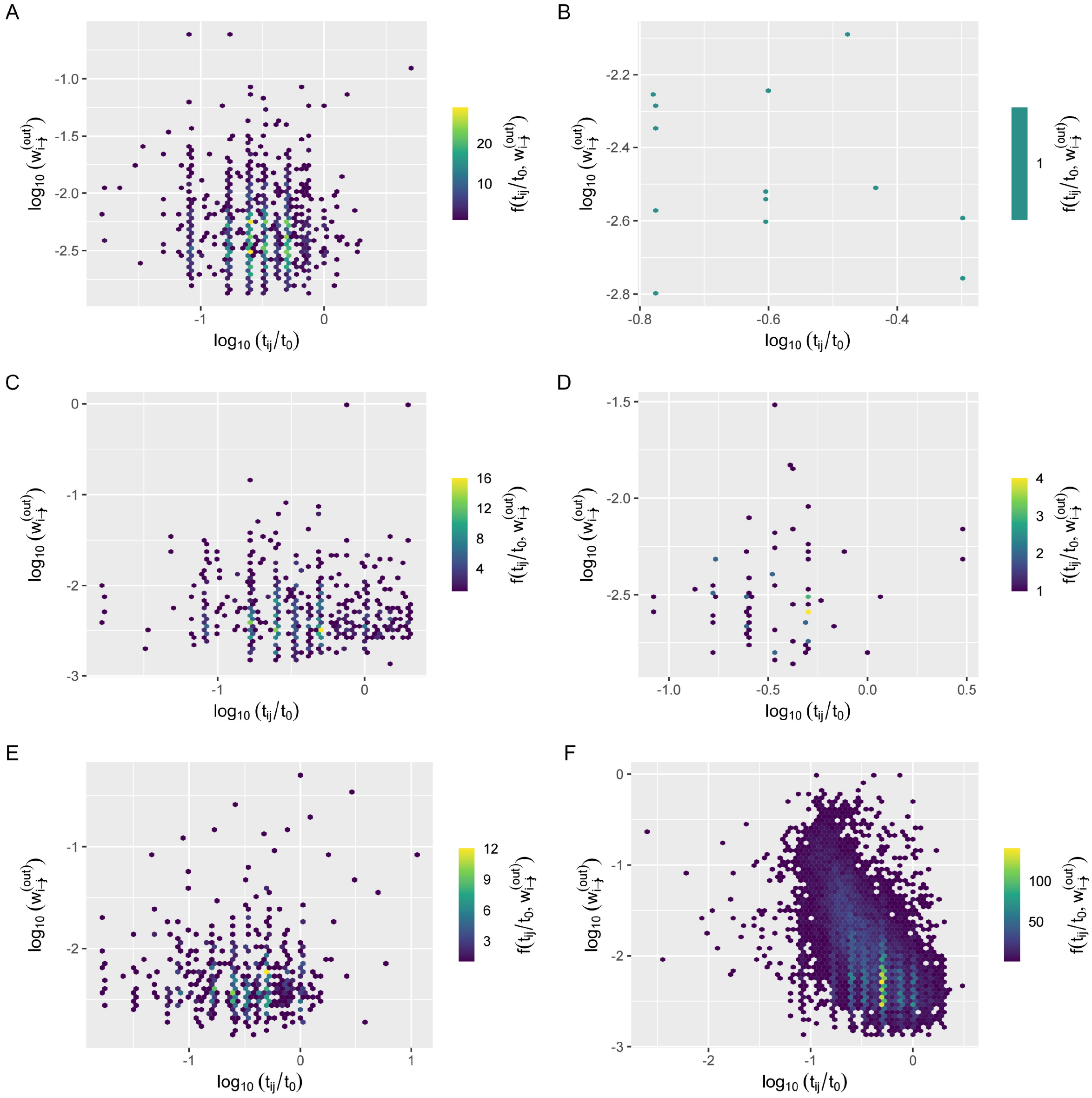

Figure 14. Time traveling according transition probabilities between zones by trip type A: (T17) Private motorbike, B: (T18) Public motorbike, C: (T19) Company motorbike, D: (T20) Private bicycle, E: (T21) Public bicycle, F: (T22) Company bicycle, G: (T23) Other, H: (T24) Walking. Graphical representations corresponding to (T18) Public motorbike and (T22)Company bicycles are not shown because they exhibited a very small number of trips.

Characterization of the topology of the mobility networks. Structural analysis

Table 12. Main structural characteristics. Number of weakly connected components in networks by trip type. (T1): Commuter trains,(T2) Intercity bus, (T3) Urban bus other municipality, (T4) Subway, (T5) Light subway, (T6) Urban bus, (T7) Rest of trains, (T8) Discretionary bus, (T9) Long-distance bus, (T10) Taxi, (T11) Driver in private car, (T12) Driver in company car, (T13) Driver or passenger in rented car without driver, (T14) Passenger in private car, (T15) Passenger in company car, (T16) Passenger in rented car with driver, (T17) Private motorbike, (T18) Public motorbike, (T19) Company motorbike (T20) Private bicycle, (T21) Public bicycle, (T22) Company bicycle, (T23) Other, (T24) Walking. No strongly connected sub-graphs were found in the networks.

**All**	**Type T1**	**Type T2**	**Type T3**	**Type T4**	**Type T5**
3	1	2	25	1	5
**Type T6**	**Type T7**	**Type T8**	**Type T9**	**Type T10**	**Type T11**
10	44	96	22	66	1
**Type T12**	**Type T13**	**Type T14**	**Type T15**	**Type T16**	**Type T17**
53	31	1	104	30	42
**Type T18**	**Type T19**	**Type T20**	**Type T21**	**Type T22**	**Type T23**
1	9	128	16	2	137
Type T24					
104					

Table 13. Degree. Trip types: (T1): Commuter trains,(T2) Intercity bus, (T3) Urban bus other municipality, (T4) Subway, (T5) Light subway, (T6) Urban bus, (T7) Rest of trains, (T8) Discretionary bus, (T9) Long-distance bus, (T10) Taxi, (T11) Driver in private car, (T12) Driver in company car, (T13) Driver or passenger in rented car without driver, (T14) Passenger in private car, (T15) Passenger in company car, (T16) Passenger in rented car with driver, (T17) Private motorbike, (T18) Public motorbike, (T19) Company motorbike (T20) Private bicycle, (T21) Public bicycle, (T22) Company bicycle, (T23) Other, (T24) Walking.

**All**	**Type T1**	**Type T2**	**Type T3**	**Type T4**	**Type T5**
136.24640	16.11412	15.08357	5.81081	35.25216	3.74194
**Type T6**	**Type T7**	**Type T8**	**Type T9**	**Type T10**	**Type T11**
31.98113	2.36364	3.78512	1.71429	4.35450	65.47813
**Type T12**	**Type T13**	**Type T14**	**Type T15**	**Type T16**	**Type T17**
4.07870	2.76923	20.84227	3.000000	2.57143	5.28447
**Type T18**	**Type T19**	**Type T20**	**Type T21**	**Type T22**	**Type T23**
2.66667	1.33333	3.75510	2.85714	1	2.88136
**Type 24**					
26.56059					

Table 14. For the comparison of the stops by zone cumulative probability distributions, the obtained $p-value$ in the Kolmogorov–Smirnov test.

	Urban bus	Light subway	Subway	Intercity bus	Commuter
Urban bus	1	0	0	0	0
Light subway	0	1	0.01	0	0
Subway	0	0.01	1	0	**0.11**
Intercity bus	0	0	0	1	0
Commuter	0	0	**0.11**	0	1

Table 15. Main structural characteristics. Number of nodes and links in *GC*. (T1): Commuter trains,(T2) Intercity bus, (T3) Urban bus other municipality, (T4) Subway, (T5) Light subway, (T6) Urban bus, (T7) Rest of trains, (T8) Discretionary bus, (T9) Long-distance bus, (T10) Taxi, (T11) Driver in private car, (T12) Driver in company car, (T13) Driver or passenger in rented car without driver, (T14) Passenger in private car, (T15) Passenger in company car, (T16) Passenger in rented car with driver, (T17) Private motorbike, (T18) Public motorbike, (T19) Company motorbike (T20) Private bicycle, (T21) Public bicycle, (T22) Company bicycle, (T23) Other, (T24) Walking.

**All**	**Type T1**	**Type T2**	**Type T3**	**Type T4**	**Type T5**
1388/94555	1034/8331	1043/7851	367/430	928/16357	31/56
**Type T6**	**Type T7**	**Type T8**	**Type T9**	**Type T10**	**Type T11**
636/10170	11/96	242/458	7/6	378/823	1349/44165
**Type T12**	**Type T13**	**Type T14**	**Type T15**	**Type T16**	**Type T17**
737/1503	26/36	1249/13016	10/9	7/823	573/44165
**Type T18**	**Type T19**	**Type T20**	**Type T21**	**Type T22**	**Type T23**
3/4	3/2	147/276	28/40	2/1	59/85
Type T24					
949/12603					

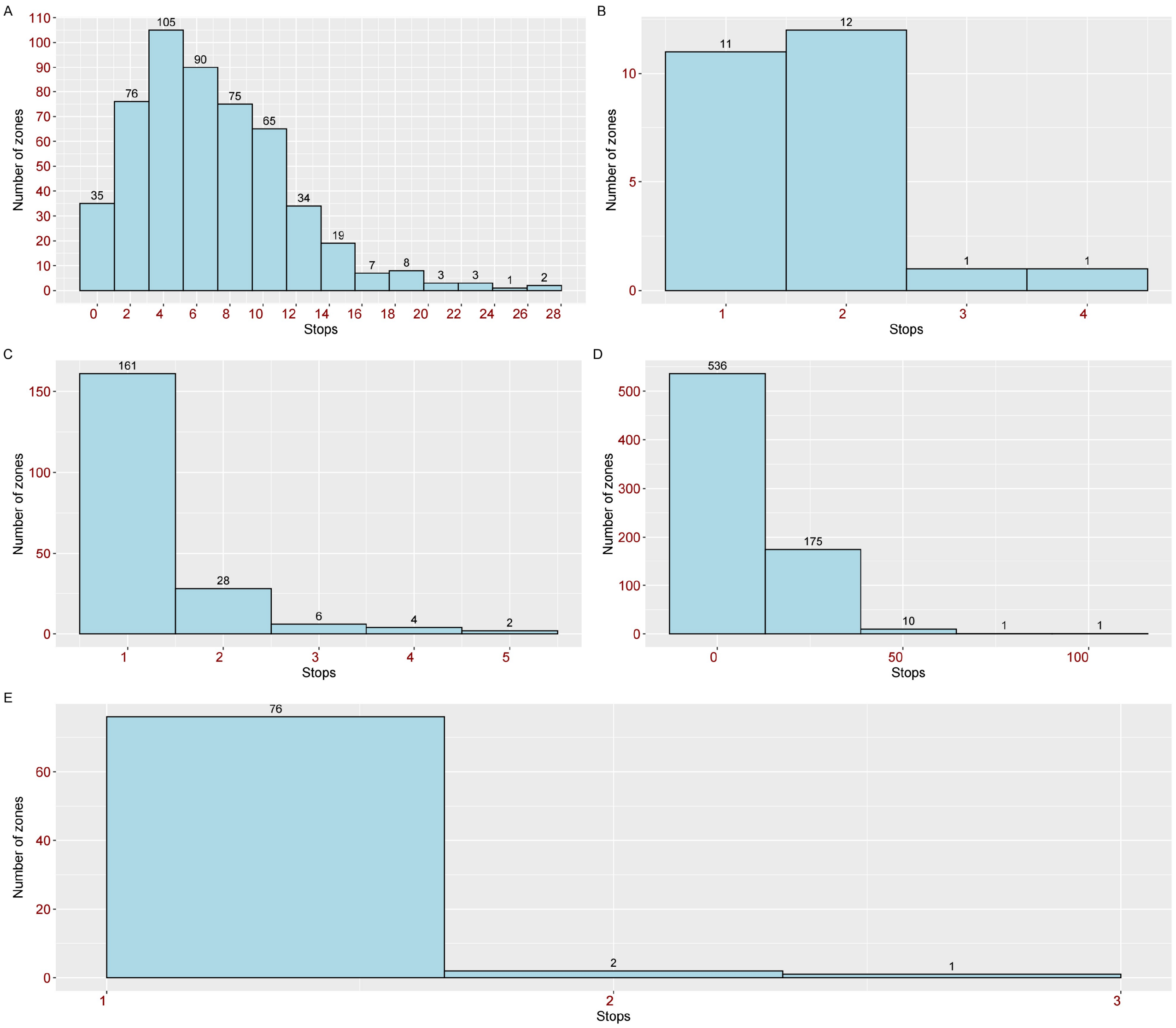

Figure 15. Histograms showing the frequency of stops by zone. A: Urban Bus, B: Light Subway, C: Subway, D: Intercity Bus, and E: Commuter Trains

Table 16. Assortativity. Trip types: (T1): Commuter trains,(T2) Intercity bus, (T3) Urban bus other municipality, (T4) Subway, (T5) Light subway, (T6) Urban bus, (T7) Rest of trains, (T8) Discretionary bus, (T9) Long-distance bus, (T10) Taxi, (T11) Driver in private car, (T12) Driver in company car, (T13) Driver or passenger in rented car without driver, (T14) Passenger in private car, (T15) Passenger in company car, (T16) Passenger in rented car with driver, (T17) Private motorbike, (T18) Public motorbike, (T19) Company motorbike (T20) Private bicycle, (T21) Public bicycle, (T22) Company bicycle, (T23) Other, (T24) Walking.

**All**	**Type T1**	**Type T2**	**Type T3**	**Type T4**	**Type T5**
0.06114	-0.10294	0.07106	-0.03371	-0.08005	-0.37610
**Type T6**	**Type T7**	**Type T8**	**Type T9**	**Type T10**	**Type T11**
0.02240	-0.71706	-0.30097	-	-0.13082	0.03400
**Type T12**	**Type T13**	**Type T14**	**Type T15**	**Type T16**	**Type T17**
-0.07556	-0.21818	0.03482	-0.50000	-0.03701	0.02312
**Type T18**	**Type T19**	**Type T20**	**Type T21**	**Type T22**	**Type T23**
-1.00000	-	-0.19416	-0.18792	-	-0.27967
**Type T24**					
0.48118					

Table 17. Betweenness. Trip types: (T1): Commuter trains,(T2) Intercity bus, (T3) Urban bus other municipality, (T4) Subway, (T5) Light subway, (T6) Urban bus, (T7) Rest of trains, (T8) Discretionary bus, (T9) Long-distance bus, (T10) Taxi, (T11) Driver in private car, (T12) Driver in company car, (T13) Driver or passenger in rented car without driver, (T14) Passenger in private car, (T15) Passenger in company car, (T16) Passenger in rented car with driver, (T17) Private motorbike, (T18) Public motorbike, (T19) Company motorbike (T20) Private bicycle, (T21) Public bicycle, (T22) Company bicycle, (T23) Other, (T24) Walking.

**All**	**Type T1**	**Type T2**	**Type T3**	**Type T4**	**Type T5**
0.00093	0.00209	0.00242	0.02236	0.00180	0.03274
**Type T6**	**Type T7**	**Type T8**	**Type T9**	**Type T10**	**Type T11**
0.00267	0.03636	0.02870	0.05714	0.00617	0.00116
**Type T12**	**Type T13**	**Type T14**	**Type T15**	**Type T16**	**Type T17**
0.00786	0.05750	0.00187	0.20833	0.08095	0.00758
**Type T18**	**Type T19**	**Type T20**	**Type T21**	**Type T22**	**Type T23**
0.33333	0.16667	0.03966	0.02951	0.02951	0.03325
**Type T24**					
0.03325					

Table 18. Diameter. Trip types: (T1): Commuter trains,(T2) Intercity bus, (T3) Urban bus other municipality, (T4) Subway, (T5) Light subway, (T6) Urban bus, (T7) Rest of trains, (T8) Discretionary bus, (T9) Long-distance bus, (T10) Taxi, (T11) Driver in private car, (T12) Driver in company car, (T13) Driver or passenger in rented car without driver, (T14) Passenger in private car, (T15) Passenger in company car, (T16) Passenger in rented car with driver, (T17) Private motorbike, (T18) Public motorbike, (T19) Company motorbike (T20) Private bicycle, (T21) Public bicycle, (T22) Company bicycle, (T23) Other, (T24) Walking.

**All**	**Type T1**	**Type T2**	**Type T3**	**Type T4**	**Type T5**
7	8	8	7	7	
**Type T6**	**Type T7**	**Type T8**	**Type T9**	**Type T10**	**Type T11**
7	3	31	4	14	7
**Type T12**	**Type T13**	**Type T14**	**Type T15**	**Type T16**	**Type T17**
22	10	8	8	4	15
**Type T18**	**Type T19**	**Type T20**	**Type T21**	**Type T22**	**Type T23**
2	2	28	8	8	17
**Type T24**					
17					

Table 19. Main structural characteristics. $k_{Max} - core$ and percentage of zones included in it by trip type. (T1): Commuter trains,(T2) Intercity bus, (T3) Urban bus other municipality, (T4) Subway, (T5) Light subway, (T6) Urban bus, (T7) Rest of trains, (T8) Discretionary bus, (T9) Long-distance bus, (T10) Taxi, (T11) Driver in private car, (T12) Driver in company car, (T13) Driver or passenger in rented car without driver, (T14) Passenger in private car, (T15) Passenger in company car, (T16) Passenger in rented car with driver, (T17) Private motorbike, (T18) Public motorbike, (T19) Company motorbike (T20) Private bicycle, (T21) Public bicycle, (T22) Company bicycle, (T23) Other, (T24) Walking.

All	Type T1	Type T2	Type T3	Type T4	Type T5
103	16 (17.70%)	14(13.16%)	8(10.81%)	37(20.80%)	3(32.26%)
**Type T6**	**Type T7**	**Type T8**	**Type T9**	**Type T10**	**Type T11**
27(13.68%)	2(45.45%)	4(15.29%)	1(100%)	5(10.05%)	50(42.18%)
**Type T12**	**Type T13**	**Type T14**	**Type T15**	**Type T16**	**Type T17**
4(19.27%)	2(84.62%)	16(39.71%)	1(100%)	2(71.42%)	5(15.53%
**Type T18**	**Type T19**	**Type T20**	**Type T21**	**Type T22**	**Type T23**
1(100%)	1(100%)	3(27.21%)	2(71.43%)	2(100%	2(76.27%)
**Type T24**					
29(8.22%)					

Table 20. Main structural characteristics. kr-core = round(0.75*kMax-core) and percentage of zones include in k-core<=kr by trip type. (T1): Commuter trains,(T2) Intercity bus, (T3) Urban bus other municipality, (T4) Subway, (T5) Light subway, (T6) Urban bus, (T7) Rest of trains, (T8) Discretionary bus, (T9) Long-distance bus, (T10) Taxi, (T11) Driver in private car, (T12) Driver in company car, (T13) Driver or passenger in rented car without driver, (T14) Passenger in private car, (T15) Passenger in company car, (T16) Passenger in rented car with driver, (T17) Private motorbike, (T18) Public motorbike, (T19) Company motorbike (T20) Private bicycle, (T21) Public bicycle, (T22) Company bicycle, (T23) Other, (T24) Walking.

All	Type T1	Type T2	Type T3	Type T4	Type T5
76-35.95%	12-68.96%	10-60.42%	6-87.84%	28-65.19%	2-67.74%)
**Type T6**	**Type T7**	**Type T8**	**Type T9**	**Type T10**	**Type T11**
20-44.50%	2-100%	3-84.71%)	1-100%	4-89.95%	38-38.25%)
**Type T12**	**Type T13**	**Type T14**	**Type T15**	**Type T16**	**Type T17**
3-80.73%	2-100%	12-39.23%	1-100%	2-100%	4-84.57%
**Type T18**	**Type T19**	**Type T20**	**Type T21**	**Type T22**	**Type T23**
1-100%	1-100%	2-72.79%)	2-100%	1-100%	2-100%
**Type T24**					
22(82.93%)					

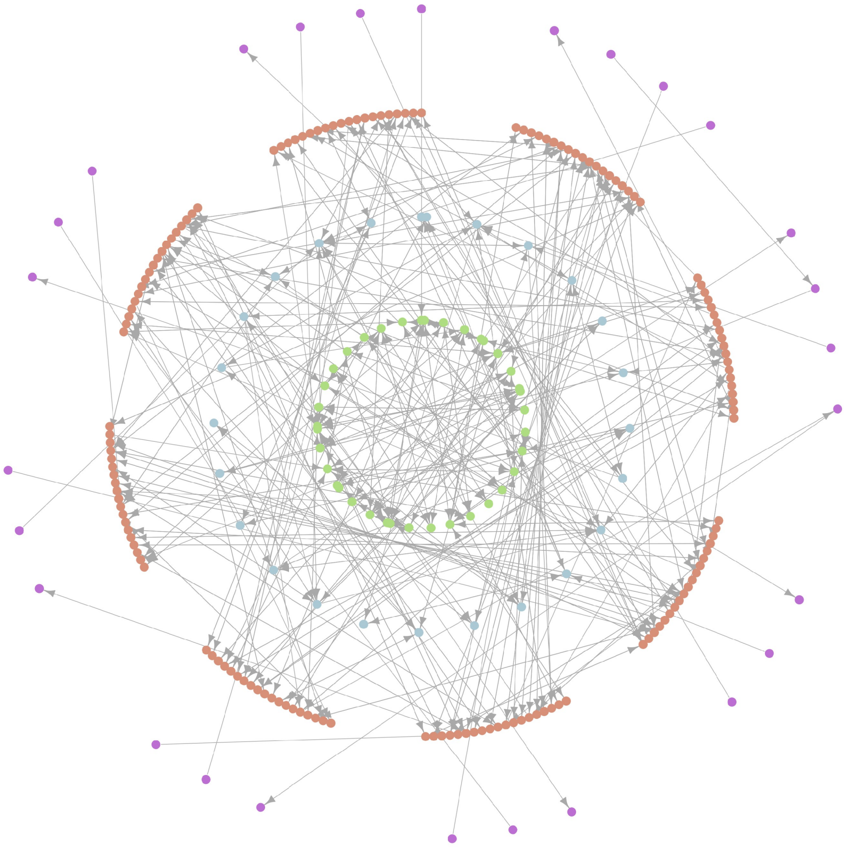

Figure 16. k-core representation for T8: Discretionary bus. Nodes drawn in the same colour are in the same k-core. Grey lines traced between pairs of nodes indicate that there is a link between them.

Table 21. For P_i^∞ of the eigenvector $\vec{P}^\infty$ with eigenvalue $\lambda = 1$ as a function of $k_i^{(in)}$, $R-squared$ corresponding to a fit polynomial regression model from degree 1 to 4, with

Degree	R-squared
1	0.50984
2	0.55254
3	0.56973
4	0.56950

Inter-zone links formation

Table 22. Importance of explanatory variables

Similarity Metric	Importance	Similarity Metric	Importance
average commute time	100	common neighb	18.07376
matrix forest	98.4185668	graph distance	10.20160
Leicht–Holme–Newman	98.41660	depressed	7.58142
random walk with restart	88.62881	cosine	7.46683
hub promoted	42.78472	cosine similarity on L+	5.97654
preferential attachment	22.94767	local path	4.28411
resource allocation	20.61072	katz	2.48136
jaccard	20.57018	Leicht–Holme–Newman	0

Table 23. Performance metrics of the model

Metric	Training+Test	Validation	Metric	Training+Test	Validation
Accuracy	0.99500	0.99476	F1	0,99502	0.99479
Sensitivity	1.00000	0.99963	Gmean	0,99499	0,99475
Specificity	0.99000	0.98990	Kappa	0.98957	0.98953
Precision	0.99010	0.98999			

Table 24. Eigenvalue (λ) The highest eigenvalue λ_{L_j} for the *GC* of all mobility networks. Trip types: (T1): Commuter trains,(T2) Intercity bus, (T3) Urban bus other municipality, (T4) Subway, (T5) Light subway, (T6) Urban bus, (T7) Rest of trains, (T8) Discretionary bus, (T9) Long-distance bus, (T10) Taxi, (T11) Driver in private car, (T12) Driver in company car, (T13) Driver or passenger in rented car without driver, (T14) Passenger in private car, (T15) Passenger in company car, (T16) Passenger in rented car with driver, (T17) Private motorbike, (T18) Public motorbike, (T19) Company motorbike (T20) Private bicycle, (T21) Public bicycle, (T22) Company bicycle, (T23) Other, (T24) Walking.

Type T1	Type T2	Type T3	Type T4	Type T5	Type T6
0.0801	0.0814	0.0809	0.1773	0.0441	0.1437
**Type T7**	**Type T8**	**Type T9**	**Type T10**	**Type T11**	**Type T12**
0.0025	0.1249	0.0563	0.0800	0.6682	0.1231
**Type T13**	**Type T14**	**Type T15**	**Type T16**	**Type T17**	**Type T18**
0.0137	0.1809	0.0261	0.0129	0.0843	0.0057
**Type T19**	**Type T20**	**Type T21**	**Type T22**	**Type T23**	**Type T24**
0.0027	-0.1508	-0.0067	0	0.1250	0.8000

Methods

Topological characterization of the mobility networks. Structural analysis

Several structural parameters were computed in the mobility networks, which were considered to be directed graphs for this topological analysis :

- $in-degree$ and $outdegree$ of a node i. $in-degree$ represented as $deg-(i)$ symbolises the number of links directed into i. The $out-degree$ of i, represented as $deg+(i)$, is the number of links directed out of i. A loop at a node participated 1 to both in-degree and out-degree.[10].
- In a directed graph, the average degree can be defined as:

$$<k>=\frac{L}{N} \tag{1}$$

where L and N, are the number of links and nodes, respectively.

- Distance, shortest path or geodesic path between two nodes i, j, which can be defined as the number of links along the shortest path joining them. In a directed graph the distance from i to j (on an $ijpath$ is commonly dissimilar from the distance from j to i (on a $jipath$). If a path of length n between i and j exist, then $A_{ik}\dots A_{lj}=1$ and $A_{ik}\dots A_{lj}=0$ otherwise. The number of paths of length n between i and j can be defined as[10]:

$$N_{ij}^{(n)} = [A^n]_{ij} \tag{2}$$

- Diameter (D), which symbolises the maximum distance between any pair of nodes i, j in the graph[10].

- Average path length/distance, $< apl >$, for a connected graph, is defined as[10]:

$$\frac{1}{2*L_{max}}\sum_{i,j\neq i} d_{i,j} \tag{3}$$

Where $d_{i,j}$ is the distance from node i to node j.

- degree assortativity, which for a directed graph can be established as[11],[12],[13]:

$$r = \sum_{jk}\frac{jk(e_{jk}-q_j^{in}q_k^{out}}{\sigma_{in}\sigma_{out}} \tag{4}$$

Where:
in, out correspond to in-degree and out-degree, respectively.
e_{jk} is the fraction of links joining a node with out-degree k to a node with in-degree j,whereby $j,k \in N$

$$q_j^{in} = \frac{(j+1)p_{j+1}^{in}}{\sum_j jp_j^{in}} = (j+1)\frac{P_{r[D_{in}=j+1]}}{E[D_{in}]} \tag{5}$$

Normalised distribution of $in-degree$, where D_{in} is the $in-degree$ of a randomly chosen node.

$$q_k^{out} = \frac{(k+1)p_{k+1}^{out}}{\sum_k kp_k^{out} = (j+1)\frac{P_{r[D_{out}=k+1]}}{E[D_{out}]}} \tag{6}$$

Normalised distribution of $out-degree$, where D_{out} is the $out-degree$ of a randomly chosen node.
$\sigma_{in}, \sigma_{out}$ standard deviation of q_j^{in}, standard deviation of q_k^{out}, respectively.
4 equation considers the correlation between a node's $out-degree$ and the adjacent node's respective $out-degree$, and the correlation between a node's $in-degree$ and the adjacent node's respective $out-degree$[14]

- The betweenness centrality of a node i in a graph (G), $bc(i)$ is[15],[16]:

$$bc(i) = \sum_{u\neq i\neq w\in G}\frac{\sigma_{u,w}(i)}{\sigma_{u,w}} \tag{7}$$

Where $\sigma_{u,w}$ is the total number of shortest paths from node u to node w and $\sigma_{u,w}(i)$ symbolises the number of those paths that pass through i.

Inter-zone links formation. Metrics related to similarities between nodes

Local metrics

Resource Allocation[17],[18] between two nodes l, m is stated as:[17],[18]

$$\sum_{u\in\Gamma(l)\cap\Gamma(m)}\frac{1}{|N(u)|} \tag{8}$$

Where $\Gamma(l)$, $\Gamma(m)$, represents the set of neighbours of l, m.

Leicht-Holme-Newman Index[19],[18] between two nodes l, m, which is defined as[19],[18]:

$$\frac{|\Gamma(l)\cap\Gamma(m)|}{k_l * k_m} \tag{9}$$

Where $\Gamma(l)$, $\Gamma(m)$, represent the set of neighbours of l, m. k_l, k_m are the degrees of l and m respectively.

Common neighbours[18] between two nodes l, m is stated as[18]

$$|\Gamma(l) \cap \Gamma(m)| \tag{10}$$

Where $\Gamma(l)$, $\Gamma(m)$, represent the set of neighbours of l, m.

Salton Index (Cosine)[20,18] between two nodes l, m, which is stated as[20,18]:

$$\sum \frac{|\Gamma(l) \cap \Gamma(m)|}{\sqrt{k_l} * \sqrt{k_m}} \tag{11}$$

k_l, k_m represent the degrees of l and m, respectively.

Cosine similarity on L+ Index[21,18]
The symmetric Laplacian matrix L of G is defined as[21,18]:

$$L = D - A \tag{12}$$

D describes a diagonal matrix, in which $d_{ll} = |D|_{ll} = a_l = \sum_{j=1}^{N} a_{lj}$. N symbolises the total number of nodes in G $L+$ indicates the Moore-Penrose pseudoinverse matrix of L, whose elements are:

$$l_{lm}^{+} = |L|^{+}{}_{lm} \tag{13}$$

The cosine similarity on L+ Index between two nodes l, m in G, is described as:

$$\frac{l_{lm}^{+}}{\sqrt{l_{lm}^{+} * l_{mm}^{+}}} \tag{14}$$

Hub Promoted Index[22,18] between two nodes l, m, which is stated as[22,18] :

$$\sum \frac{|\Gamma(l) \cap \Gamma(m)|}{min(k_l, \ k_m)} \tag{15}$$

Where $\Gamma(l)$, $\Gamma(m)$, represent the set of neighbours of l, m. k_l, k_m are the degrees of the nodes l, m; (k_l, k_m) is the minimum of k_l and k_m.

Jaccard Coefficient[23,18] between two nodes l, m, which is defined as[23,18]:

$$\sum \frac{|\Gamma(l) \cap \Gamma(m)|}{|\Gamma(l) \cup \Gamma(m)|} \tag{16}$$

where $\Gamma(l)$ and $\Gamma(m)$ represent the set of neighbours of l and m.

Hub Depressed Index[22,18] between two nodes l, m, which is defined as[22,18]:

$$\frac{|\Gamma(l) \cap \Gamma(m)|}{max(k_l, \ k_m)} \tag{17}$$

Where $\Gamma(l)$, $\Gamma(m)$, symbolise the set of neighbours of l, m; k_l, k_m are the degrees of l and m; (k_l, k_m) indicate the maximum of k_l and k_m.

Preferential Attachment Index[10,18] between two nodes l, m, which is defined as[10,18]:

$$k_l * k_m \tag{18}$$

Where: k_l, k_m are the degrees of l and m.

Sørensen Index[24,18] between two nodes l, m, which can be stated as:[24,18]:

$$\frac{2 * |\Gamma(l) \cap \Gamma(m)|}{k_l + k_m} \tag{19}$$

$\Gamma(l)$, $\Gamma(m)$, symbolise the set of neighbours of l, m; and k_l, k_m represent the degrees of l and m.

Quasilocal methods

Graph distance[18] between two nodes l, m, which can be described as[18]:

$$\infty \quad if \quad l = m \tag{20}$$

$$0 \quad if \quad l \quad and \quad m \quad are \quad not \quad linked \tag{21}$$

$$\frac{1}{p_{lm}} \quad in \quad other \quad cases \tag{22}$$

$$\tag{23}$$

$p_{lm} = min(l : path_{lm}^{<lp>} \; exists)$ symbolises the length of the shortest path joining l and m.

Local Path Index[17,18] A LPI matrix is described as[17,18]:

$$LPI = A^2 + \alpha^1 A^3 + \alpha^2 A^4 + \ldots + \alpha^{n-2} A^n \tag{24}$$

Where: $n > 2$, A represents the adjacency matrix, α is a free parameter.
The Local Path Index between two nodes l, m, is established by the component lm of the LPI matrix.

Global methods

Average Commute Time Index
[21,18] between two nodes l, m, which is stated as[21,18]:

$$\frac{1}{l_{ll}{}^{+} + l_{jj}{}^{+} - 2 * l_{lm}{}^{+}} \tag{25}$$

where l and m represent nodes, $l_{ll}{}^{+}$, $l_{mm}{}^{+}$, $l_{lm}{}^{+}$ symbolise the associated entries in Laplacian Matrix, L^{+}.

Katz Index Index[25,18] between two nodes l, m, which are defined as[25,18]:
A sum of the products of all path lengths in G, and β is a free parameter which is utilised to manage the path weight. Therefore, it can be computed as:

$$\sum_{l=1}^{\infty} \beta^{pl} \; |paths_{lm} \;|^{<pl>} \tag{26}$$

$|paths_{lm} \;|^{<pl>}$ represent the set of all paths with length pl joining l and m. β symbolises the free parameter. The sum converges at the time when β is lower than the reciprocal of the largest eigenvalue of the adjacency matrix, A, of G.

L+ directly Index[21,18] between two nodes l,m corresponds to the component l_{lm} + of L^{+}.

Matrix Forest Index[26,18] This index is defined through a similarity matrix which can stated as[26,18]:

$$\frac{1}{I - L} \tag{27}$$

Where: I symbolises the identity matrix, L represents the Laplacian matrix of G. L which can be stated as: $L = D - A$, D and A representing the degree and the adjacency matrixes of G, respectively.
Random Walk with Restart[27,18] between two nodes l, m, which can be stated as[27,18]:

$$q_{lm} + q_{ml} \tag{28}$$

q_{ij} symbolises the probability with which a random walker starting at node i will come back to node i itself visiting a random neighbour.

Inter-zone links formation. Performance metrics of the model

To evaluate the model the following performance metrics are used:

- Accuracy, which is described as[18]:

$$Accuracy = \frac{TP+TN}{TP+TN+FP+FN} \tag{29}$$

  Where: TP, TN, FP, and FN represent the true positives, the true negatives, the false positives, and the false negatives.

- Sensitivity or Recall, which is stated as[18]:

$$Sensitivity = \frac{TP}{TP+FN} \tag{30}$$

- Specificity or Selectivity, which is defined as[18]:

$$Specificity = \frac{TN}{TN+FP} \tag{31}$$

- Precision, which is stated as:

$$Precision = \frac{TP}{TP+FP} \tag{32}$$

- F1 which is defined as:

$$F1 = 2 * \frac{Precision * Recall}{Precision + Recall} \tag{33}$$

- Geometric mean, which is stated as:

$$GMean = \sqrt{Sensitivity * Specificity} \tag{34}$$

- Area under Curve (AUC). A receiver Operating Characteristic curve (ROC) symbolises a probability curve in which each point is equivalent a true positive proportion (TPR)/ false positive rate (FPR) pair representing one decision threshold. TPR and FPR are stated as[18]:

$$TPR = \frac{TP}{TP+TN} \tag{35}$$

$$FPR = \frac{FP}{TP+TN} \tag{36}$$

  ROC(t) is the function describing the ROC curve. The (AUC) is described as[18]:

$$AUC = \int_0^1 ROC(t)\ dt \tag{37}$$

- Kappa, which is defined as[28]:

$$kappa = \frac{Predicted\ accuracy - Expected\ accuracy}{1 - Expected\ accuracy} \tag{38}$$

  Where:

$$Predicted\ accuracy = \frac{TP+TN}{TP+FP+TN+FN} \tag{39}$$

$$Expected\ accuracy = \frac{TP+FP}{TP+FP+TN+FN} * \frac{TP+FN}{TP+FP+TN+FN} * \frac{FN+TN}{TP+FP+TN+FN} * \frac{FP+TN}{TP+FP+TN+FN} \tag{40}$$

Applicability of the used methods in this research

The methodology of this research can be applied to the mobility examination in any city (twin cities, megacities, megalopolis, etc.), where a zonal division has been established, even in those where non-motorised transport use is relevant (Ghent or Amsterdam).

Zoning can be based on criteria of socio-economic homogeneity, urban planning and accessibility to transport infrastructure. The following data are required: origin and destination zones, travel distances, trip duration, priority travel mode, priority reason for the trip, start and end times of the journey, and public transport stops by zones.

In particular, the major flows (hotspot - non-hotspots (HN) , non-hotspot (NH) - hotspots, hotspots- hotspots (HH), non-hotspot - non-hotspot (NN)), the network topology as well as the link formation process can be analysed.

This research may help to better understand some of the aspects of human mobility in the Madrid Community. Knowledge of travel habits can be useful to achieve a better match between public transport supply and demand. Specifically, for the major flows HH, HN, and NH, the planning of public transport between zones at peak travel times should be reviewed, considering the high percentage of private vehicle use.

The much greater use of private vehicles to the detriment of public transport, which has occurred systematically in recent years[29], has led to some structural changes in the street network, particularly in the city of Madrid[30]. The Madrid Community has also drawn up a Strategic Plan for Sustainable Mobility for the period 2013-2025[29], being one of its purposes reducing the environmental impact of transport. Specific initiatives have been carried out such as BiciMad[31] or CarSharing[32]. The Madrid City Hall is also reviewing and extending the transport network with safe routes, aiming to integrate more sustainable modes of transport with traditional motorised commuting. The goal is to increase cycling to 5% of trips by 2025[33].

According to this research, a relationship exists between certain journeys which are made on foot alongside those made through other motorised forms in which the distances are similar. The above suggests that some additional actions from the institutions could be taken to promote greater public awareness of the importance of using more sustainable transport. This is particularly relevant since a significant increase in the number of trips is expected in the coming years, given the availability of residential and productive land for development in some municipalities.

Analogously, in municipalities with high population density and a significant proportion of hotspots the use of shared autonomous vehicles can gradually transform public transport. Fixed schedules and routes involving smaller vehicles with dynamic route assignments can, if demand is adequate, directly link points of origin and destination. Thus, the number of travel stages required to reach a destination from an origin is reduced[34].

An analogous study carried out in this research considering several cities by country, or either at a continental or even global level can be performed, which would provide a better understanding of human mobility. It would also be possible to detect how sustainable mobility is in those cities and take action from one and apply it to another.

References

1. TCM. Encuesta de mobilidad de la Comunidad de Madrid 2018. Documento síntesis. Available online at: https://www.crtm.es/media/712934/edm18_sintesis.pdf, last accessed on 23.06.2022.
2. Powered by CRTM. Datos abiertos CRTM. Consorcio Regional de Transportes de Madrid CRTM. EDM2018VIAJES. Available online at: https://datos.crtm.es/documents/6afd4db8175d4902ada0803f08ccf50e/about., last accessed on 23.06.2022.
3. Powered by CRTM. Datos abiertos CRTM. Consorcio Regional de Transportes de Madrid CRTM. ZONIFICACIONZT1259. Available online at: https://datos.crtm.es/documents/6afd4db8175d4902ada0803f08ccf50e/about., last accessed on 23.06.2022.
4. Powered by CRTM. datos abiertos CRTM. Available online at: https://data-crtm.opendata.arcgis.com/, last accessed on 23.06.2022.
5. MTMyA. Ministerio de Transportes, Movilidad y Agenda Urbana. Sistema de información urbana. Available online at: https://www.mitma.gob.es/portal-del-suelo-y-politicas-urbanas/sistema-de-informacion-urbana/sistema-de-informacion-urbana-siu, last accessed on 18.11.2022.
6. INE. Insituto Nacional de Estadística. Cifras oficiales de población resultantes de la revisión del padrón municipal a 1 de enero. Available online at:https://www.ine.es/jaxiT3/Tabla.htm?t=2881&L=0, last accessed on 18.11.2022.
7. Ministerio de Transportes, Movilidad y Agenda Urbana. Sistema de información urbana. Available online at: https://www.mitma.gob.es/portal-del-suelo-y-politicas-urbanas/sistema-de-informacion-urbana/sistema-de-informacion-urbana-siu, last accessed on 23.11.2022.
8. Available online at: http://www.tesintegra.net/maps/geojson/es/municipios/, last accessed on 23.11.2022.

9. Instituto Nacional de Estadística. Cifras oficiales de población resultantes de la revisión del padrón municipal a 1 de enero. Available online at: https://www.ine.es/jaxiT3/Tabla.htm?t=2881&L=0, last accessed on 23.11.2022.
10. Barabási, A., Towlson, E., Ruf, S., Danziger, M. & Shekhtman, L. Network science class 2: graph theory (ch2). Available online at: https://www.ic.unicamp.br/~meidanis/courses/mo412/2020s1/slides/graph/Class2_2020_theory.pdf, last accessed on 23.06.2022.
11. Newman., M. E. J. The structure and function of complex networks. *SIAM Rev.* **45** (2003).
12. Newman., M. E. J. Mixing patterns in networks. *Phys. Rev. E* **67** (2003).
13. Newman., M. E. J. Assortative mixing in networks. *Phys. Rev. Lett.* **89** (2002).
14. Noldus, R. & Mieghem, P. Assortativity in complex networks. *J. Complex Networks 2015* DOI: https://doi.org/10.1093/comnet/cnv005 (2015).
15. Estrada, E., Higham, D. & Hatano, N. Communicability betweenness in complex networks. *Phys. A: Stat. Mech. its Appl.* **388**, DOI: https://doi.org/10.1016/j.physa.2008.11.011 (2009).
16. Mouronte-López, M. L. Modeling the public transport networks: a study of their efficiency. *Complexity* **2021**, 1–19, DOI: https://doi.org/10.1155/2021/3280777 (2021).
17. Zhou, T., Lü, L. & Yi-Cheng Zhang, Y.-C. Predicting missing links via local information. *The Eur. Phys. J. B* **71**, 623–630, DOI: https://doi.org/10.1140/epjb/e2009-00335-8 (2009).
18. Mouronte, M. L. Modeling the public transport networks: a study of their efficiency. *Complexity* **2021**, 1–19, DOI: https://doi.org/10.1155/2021/3280777 (2021).
19. Leicht, E. A., Holme, P. & Newman, M. E. J. Vertex similarity in networks. *Phys. Rev. E* **73**, DOI: https://doi.org/10.1103/physreve.73.026120 (2006).
20. Salton, G. & McGill, M. J. *Introduction to modern information retrieval* (McGraw-Hill, Inc., 1986).
21. Fouss, F., Pirotte, A., Renders, J.-M. & Saerens, M. Random-walk computation of similarities between nodes of a graph with application to collaborative recommendation. *IEEE Transactions on Knowl. Data Eng.* **19**, 355–369, DOI: https://doi.org/10.1109/TKDE.2007.46 (2007).
22. Ravasz, E., Somera, A. L., Mongru, D. A., Oltvai, Z. N. & Barabási, A. L. Hierarchical organization of modularity in metabolic networks. *Science* **297**, 1551–1555, DOI: https://doi.org/10.1126/science.1073374 (2002).
23. Jaccard, P. Étude comparative de la distribution florale dans une portion des Alpes etdes Jura. *Bull. del la Société Vaudoise des Sci. Naturelles* **37**, 547–579 (1901).
24. A., S. T. A method of establishing groups of equal amplitude in plant sociology based on similarity of species content and its application to analyses of the vegetation on danish commons. *Biol. Skrifter* **5**, 1–34 (1948).
25. Katz, L. A new status index derived from sociometric analysis. *Psychometrika* **18**, 39–43 (1953).
26. Jia-Yu, P., Hyung-Jeong, Y., Christos, F. & Pinar, D. Automatic multimedia cross-modal correlation discovery. In *Proceedings of the Tenth ACM SIGKDD International Conference on Knowledge Discovery and Data Mining*, KDD '04, 653–658, DOI: https://doi.org/10.1145/1014052.1014135 (Association for Computing Machinery, New York, NY, USA, 2004).
27. Brin, S. & Page, L. The anatomy of a large-scale hypertextual web search engine. *Comput. Netw. ISDN Syst.* **30**, 107–117, DOI: 10.1016/S0169-7552(98)00110-X (1998).
28. Mirza, A., Asghar, S. & Noor, M. A classification model for class imbalance dataset using genetic programming. *IEEE Access* **7**, 71013–71037, DOI: http://dx.doi.org/10.1109/ACCESS.2019.2915611 (2019).
29. TCM. Plan estratégico de movilidad sostenible de la Comunidad de Madrid 2013 - 2025. Available online at: https://mcyt.educa.madrid.org/uploads/Anexos_S4/Anexo%205.5.%20Plan%20Estrat%C3%A9gico%20de%20Movilidad%20Sostenible%202013-2025.pdf, last accessed on 18.11.2022.
30. DOM. Estado de la movilidad de la ciudad de Madrid. Available online at: https://transparencia.madrid.es/FWProjects/transparencia/Movilidad/Trafico/InformesMovilidad/Ficheros/InformeMovilidad2020.pdf, last accessed on 18.11.2022.
31. Hall, M. C. BiciMad. Available online at: https://www.bicimad.com/, last accessed on 18.11.2022.
32. Hall, M. C. Portal web del Ayuntamiento de Madrid. Coches (carsharing). Available online at: https://www.madrid.es/portales/munimadrid/es/Inicio/Movilidad-y-transportes/Direcciones-y-telefonos/Servicios-de-movilidad-compartida-sin-base-fija/

?vgnextfmt=default&vgnextoid=65bd97f7dec76610VgnVCM1000001d4a900aRCRD&vgnextchannel=262f8fb9458fe410VgnVCM1000000b205a0aRCRD&idCapitulo=10804493, last accessed on 18.11.2022.

33. MCH. Portal web del Ayuntamiento de Madrid. Las 30 medidas. Available online at: https://www.madrid.es/portales/munimadrid/es/Inicio/Medio-ambiente/Las-30-medidas?vgnextfmt=default&vgnextoid=0590b83903a50610VgnVCM1000001d4a900aRCRD&vgnextchannel=3edd31d3b28fe410VgnVCM1000000b205a0aRCRD, last accessed on 18.11.2022.

34. TCM. Plan especial de mejora de la red pública y ordenación pormenorizada del intercambiador de trasportes de Conde de Casal. Available online at: https://www.comunidad.madrid/transparencia/sites/default/files/regulation/documents/documento_grafica._planos.pdf, last accessed on 18.11.2022.
